# Large-scale analysis of temporal gene expression variation in peripheral blood

**DOI:** 10.1038/s41467-026-73218-6

**Published:** 2026-05-29

**Authors:** Neha Mishra, Franziska Kimmig, Doris Vandeputte, Valentina Talevi, Lindsey De Commer, Chloe Verspecht, Arnau Vich Vila, Julia S. El-Sayed Moustafa, Lukasz Kreft, Alexander Botzki, Youssef El Darzi, Sebastian Proost, Lindsay Devolder, Dongmeng Wang, Joana P. Bernardes, N. Ahmad Aziz, SYSCID consortium, Neha Mishra, Doris Vandeputte, Konrad Aden, Vibeke Andersen, Aggelos Banos, George Bertsias, Marc Beyer, Johanna I. Blase, Dimitrios Boumpas, Paraskevi Christofidou, Axel Finckh, Gilles Gasparoni, Michel Georges, Wei Gu, Robert Häsler, Stephan Huthmacher, Mohamad Jawhara, Amy Kenyon, Christina Kratsch, Roland Krause, Gordan Lauc, Paul A. Lyons, Massimo Mangino, Eoin F. McKinney, Gioacchino Natoli, Karl Nordström, Marek Ostaszewski, Silja H. Overgaard, Marija Pezer, Souad Rahmouni, Benedikt Reiz, Elisa Rosati, Despina Sanoudou, Venkata Satagopam, Reinhard Schneider, Jonas Schulte-Schrepping, Prodromos Sidiropoulos, Kenneth G. C. Smith, Signe B. Sørensen, Timothy Spector, Aleksandar Vojta, Jörn Walter, Stefanie Warnat-Herresthal, Vlatka Zoldoš, Andre Franke, Stefan Schreiber, Emmanouil T. Dermitzakis, Joachim L. Schultze, Jeroen Raes, Philip Rosenstiel, Andre Franke, Stefan Schreiber, Emmanouil T. Dermitzakis, Sara Vieira-Silva, Gwen Falony, Kerrin S. Small, Monique M. B. Breteler, Joachim L. Schultze, Jeroen Raes, Philip Rosenstiel

**Affiliations:** 1 Institute of Clinical Molecular Biology, Christian-Albrechts-University Kiel and University Hospital Center Schleswig-Holstein, Kiel, Germany; 2 Laboratory of Molecular Bacteriology, Department of Microbiology and Immunology, Rega Institute, KU Leuven, Leuven, Belgium; 3 Center for Microbiology, VIB-KU Leuven, Leuven, Belgium; 4 AIMS lab, Faculty of Medicine and Pharmacy, Vrije Universiteit Brussel, Brussels, Belgium; 5 Laboratory of Microbiology, Department of Biochemistry and Microbiology, Ghent University, Ghent, Belgium; 6 Population Health Sciences, German Centre for Neurodegenerative Diseases (DZNE), Bonn, Germany; 7 Department of Twin Research and Genetic Epidemiology, King’s College London, London, UK; 8 VIB Bioinformatics Core, VIB, Ghent, Belgium; 9 VIB Technology Training, VIB, Ghent, Belgium; 10 Centre of Neurology, Clinic for Parkinson, Sleep and Movement Disorders, University Hospital Bonn, University of Bonn, Bonn, Germany; 11 Institute for Medical Biometry, Informatics and Epidemiology (IMBIE), University Hospital Bonn, University of Bonn, Bonn, Germany; 12 Department of General Internal Medicine I, University Hospital Schleswig-Holstein, Kiel, Germany; 13 Institute of Genetics and Genomics of Geneva (iGE3), University of Geneva, Geneva, Switzerland; 14 Department of Genetic Medicine and Development, Faculty of Medicine, University of Geneva, Geneva, Switzerland; 15 Swiss Institute of Bioinformatics, Geneva, Switzerland; 16 Institute of Medical Microbiology and Hygiene and Research Centre for Immunotherapy (FZI), University Medical Centre of the Johannes Gutenberg University, Mainz, Germany; 17 Institute of Molecular Biology (IMB), Mainz, Germany; 18 Institute for Quantitative and Computational Biosciences (IQCB), Johannes Gutenberg University, Mainz, Germany; 19 Host-Microbe Interactomics Group, Wageningen University, Wageningen, The Netherlands; 20 Systems Medicine, German Center for Neurodegenerative Diseases (DZNE), Bonn, Germany; 21 Genomics & Immunoregulation, Life & Medical Sciences (LIMES) Institute, University of Bonn, Bonn, Germany; 22 PRECISE Platform for Single Cell Genomics and Epigenomics, DZNE and University of Bonn, Bonn, Germany; 23 Institute of Molecular Medicine, University of Southern Denmark, Odense, Denmark; 24 Center of Clinical, Experimental Surgery and Translational Research, Biomedical Research Foundation Academy of Athens, Athens, Greece; 25 Institute of Molecular Biology and Biotechnology, Foundation for Research and Technology – Hellas (FORTH), Heraklion, Greece; 26 QMUL Centre for Epigenetics, Queen Mary University of London, London, UK; 27 Geneva University Hospital, Rheumatology Division, Geneva, Switzerland; 28 Department of Genetics, University of Saarland, Saarbrücken, Germany; 29 Unit of Animal Genomics, GIGA Research Center, Faculty of Veterinary Medicine, University of Liège, Liège, Belgium; 30 Luxembourg Centre for Systems Biomedicine, University of Luxembourg, Esch-sur-Alzette, Luxembourg; 31 Comma Soft AG, Bonn, Germany; 32 Department of Experimental Oncology, IEO, European Institute of Oncology IRCCS, Milan, Italy; 33 Genos Glycoscience Research Laboratory, Zagreb, Croatia; 34 Cambridge Institute of Therapeutic Immunology and Infectious Disease, Jeffrey Cheah Biomedical Centre, Cambridge Biomedical Campus, Cambridge, UK; 35 Department of Biology, Faculty of Science, University of Zagreb, Zagreb, Croatia

**Keywords:** Genome-wide analysis of gene expression, Gene expression, Gene expression profiling

## Abstract

Transcriptomic profiling of peripheral blood offers a promising, non-invasive approach for disease diagnosis and monitoring. However, its clinical translation is hindered by limited knowledge of the natural temporal variation. Here, we present a comprehensive reference map of longitudinal transcriptomic variability, based on RNA-sequencing of 333 healthy individuals sampled at three time points over six months. We find that 85% of genes and 99% of transcripts exhibit greater intra-individual than inter-individual variation, primarily driven by dynamic regulation of housekeeping pathways. In contrast, immune-related transcripts –particularly those linked to T and B cell activity– are strikingly stable over time. Gene expression levels drive inter-individual differences, while splicing variation contributes more to intra-individual fluctuation. In an independent twin cohort (148 monozygotic, 166 dizygotic), genes with high inter-individual variability show greater heritability, suggesting genetic control of steady-state expression. By integrating extensive clinical and environmental data, we trace temporal expression changes to genetic, compositional, and external factors, and identify robust seasonal and sex-specific signatures. These findings were validated in a third, cross-sectional cohort of 3,480 individuals. The observed temporal variation patterns have important implications for cohort-based transcriptomic analyses, as they may limit discovery and reproducibility of expression quantitative trait loci and increase the risk of spurious associations in cross-sectional studies. This resource provides a critical baseline for distinguishing disease-associated transcriptomic changes from normal physiological variation, advancing the reliability of blood-based biomarkers in clinical practice.

## Abstract (web summary)

Peripheral blood transcriptomics is emerging as a powerful, non-invasive tool for disease monitoring. Here, the authors provide a comprehensive reference catalogue of longitudinal RNA variability, defining individual healthy dynamics and improving interpretation of blood-based biomarkers.

## Introduction

High-throughput RNA sequencing has revolutionized our understanding of gene expression patterns associated with health and disease^1,2^. Peripheral blood is the most accessible and widely used human biomaterial in clinical research and practice^3,4^. Blood-based RNA biomarkers as a systemic proxy of pathological changes may aid in diagnosis, prognosis, and therapeutic decision-making^5^. The potential has been substantiated in a plethora of human diseases including monogenic syndromes^6,7^, cancer^8–10^, inflammatory disorders^11–19^, metabolic syndrome^20^ and neurodegeneration^21–23^. During the SARS-CoV2 pandemic, RNA-based analyses from peripheral blood were among the first published molecular studies^11,14,24–27^, which delineated mechanisms and biomarkers for a severe disease course and complications, e.g., thromboembolic events or respiratory failure. Prior longitudinal transcriptomic studies have yielded important insights into gene expression dynamics in specific cell types (e.g., platelets^28^) and in the context of early chronic disease manifestation (e.g., type II diabetes^29^) and seasonal variation^30^. However, the establishment of a robust reference catalogue of temporal RNA variation in a population-based cohort of healthy individuals is crucial to benchmark such individual components of marker signatures against normal physiological variability. Inter-individual differences in gene expression have been rigorously studied^31–35^ through genetic association approaches, particularly eQTL mapping and heritability analyses, which have been instrumental in identifying regulatory variants and linking genotype to transcriptional phenotypes. At the same time, these studies indicate that genetic variation explains only a portion of total expression variability, highlighting additional, substantial contributions from dynamic and context-dependent regulatory mechanisms^36^. Understanding the magnitude and potential sources of longitudinal variation is therefore essential for differentiating between true disease-associated alterations and natural fluctuations before they can be introduced into the clinical setting.

Here, we address this gap by establishing a comprehensive reference of inter- and intra-individual transcriptomic variation in a Central European population cohort of 333 individuals, using longitudinal peripheral blood RNA sequencing over one year. Using rigorous quality control measures and standardized data analysis pipelines, we compiled a dataset encompassing diverse demographic and clinical characteristics, including age, sex, diet, seasonality, and diverse clinical parameters. We captured the dynamics of gene expression patterns, differential transcript usage and identified key genes and pathways associated with high intra- and/or inter-individual variation. We delineate the contribution of heritability of gene expression to these observations using blood RNAseq data from an independent cohort of 314 mono- or dizygotic twins. We determined sex-specific patterns of intra- and inter-individual variation and confirm our findings in an independent cohort of 3480 individuals. Our blood reference catalogue may thus serve as a framework for future studies, enabling researchers to compare their findings against a well-defined healthy transcriptomic landscape.

## Results

### Study design

To delineate the physiological temporal variation of gene expression in peripheral blood at population-level, we conducted a large-scale sampling effort in a confined geographic region (Flanders, Belgium). The collection was part of a larger population study (Flemish Gut Flora Project^37^) and was accompanied by exhaustive phenotyping through web-based surveys, standardized medical history and health evaluations conducted by general medical practitioners, as well as comprehensive clinical laboratory profiling (Supplementary Fig. 1a–c and Supplementary Data 1). From this cohort, we selected samples from 333 individuals (96% born in Belgium) at 3 different time points (3 months apart), resulting in a total of 999 samples. Samples from 267 individuals were collected between February and August (Cohort A) and those from 66 more individuals were collected between August and February (Cohort B) (Supplementary Fig. 1a and b). Details on the cohorts, including clinical and laboratory characteristics are included as supplement (Supplementary Data 1). Incorporating a balanced diversity of age, health conditions, immunological events (e.g., self-limiting infections, hay fever, vaccination) and other lifestyle factors, the cohort is anticipated to reflect the typical blood transcriptome variation in a Western European population (Supplementary Fig. 1c).

RNA sequencing (RNA-seq) was performed to a median depth of 26 million paired reads/sample using a stranded mRNA protocol. The resulting data provide the so far deepest longitudinal survey of individual-specific gene expression from peripheral blood samples, enabling a comprehensive resource for studying the impact of time (scale of months) and the effects of a multitude of clinical covariates on physiological gene expression levels.

### Sample donor and cellular blood composition are major sources of variation in gene-level expression data

**Fig. 1:**
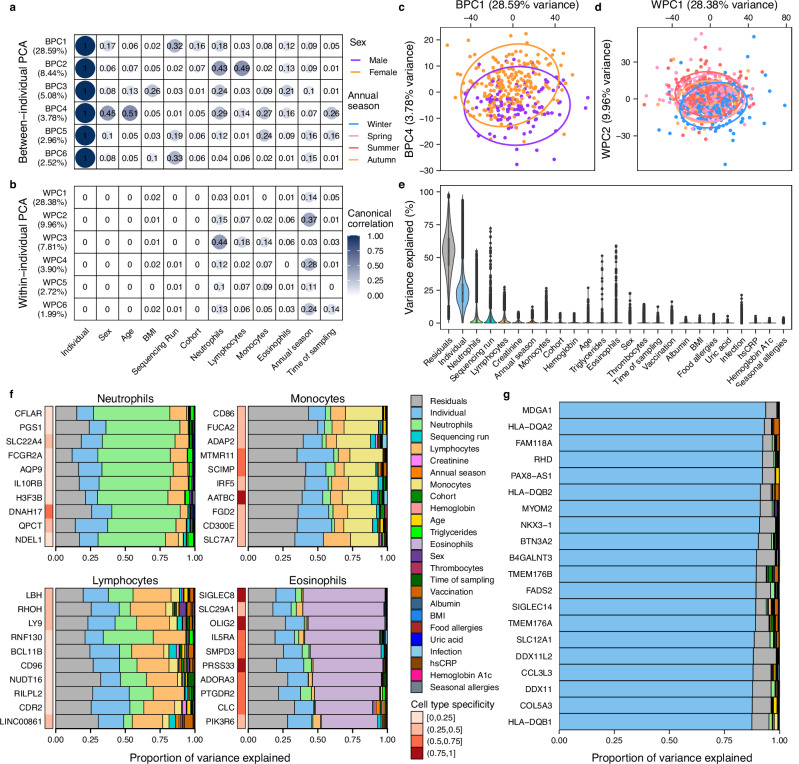
Quantitative partitioning of variance in gene expression. **a, b**, Canonical correlations between the first six between-individual (BPCs; **a**) and within-individual principal components (WPCs; **b**) and sample parameters. Colour intensity and circle size indicate the magnitude of correlation. **c**, **d** Between- and within-individual PCA plots for all samples based on the expression of genes passing the filtering cutoff (*n* = 14,166, see “Methods”), coloured by biological sex (**c**) and annual season (**d**). **e** Distribution of the proportion of variance in gene expression explained by clinical and technical parameters across genes (*n* = 13,702). Each data point represents a gene. Violin plots show the distribution of the data, with the width representing kernel density. The centre line indicates the median, the box denotes the interquartile range (IQR), whiskers extend to 1.5× the IQR, and points represent outlier genes beyond the whiskers. **f**, **g** Gene-level variance explained by each parameter for the top genes ranked by variance attributable to blood cell counts (**f**; top 10 genes) and individual identity (**g**; top 20 genes). Vertical heatmap in (**f**) shows cell-type specificity scores deduced from independent scRNAseq data. Source data are provided as a Source Data file.

For a global overview of inter- and intra-individual population-scale blood gene expression variation, we first performed multilevel principal component analysis (multiPCA) using gene-level expression estimates^38^. This method decomposes expression variance into between- and within-individual components, enabling separate assessment of temporal and between-subject effects. The first six principal components of between- and within-individual expression accounted for over 50% of the respective variation (Fig. 1a and b). Between-individual principal components (BPCs) primarily reflected stable individual-specific and technical factors, including age and sex (BPC4), sequencing run (BPCs 1 and 6), cohort (BPC1), diurnal time of sampling (BPCs 4 and 5), and variation in blood cell counts (BPCs 1–5; Fig. 1a and c and Supplementary Fig. 2a). In contrast, the within-individual principal components (WPCs) were dominated by temporal effects, with seasonal variation contributing strongly to multiple components (WPCs 1, 2, 4, and 6; Fig. 1b and d and Supplementary Fig. 2a).

To quantify the underlying sources of variation in gene-level expression, we performed variance partition analysis with available technical and phenotypic variables for each sample (Supplementary Data 1 and 2). The sample donor (“individual”; 24.4%), blood cellular composition (neutrophils, lymphocytes, monocytes, eosinophils; 9.7% combined), creatinine (1%), age (0.66%), and sex (0.45%) emerged as major known contributors to overall variation (Fig. 1e and Supplementary Fig. 2b). Other noteworthy known sources included technical factors such as sequencing run (3.5%), annual season (1.1%), cohort membership (cohort A vs. cohort B; 0.85%) and diurnal sampling time (0.37%) while a large part of the variation (“residuals”; 54.1%) remained unexplained by the investigated parameters (Fig. 1e and Supplementary Fig. 2b). To further reduce the impact of known technical variation, we employed the factor analysis-based method RUVseq^39^ to capture and combine this variation in synthetic factors (see “Methods”), which were used as covariates in the subsequent analyses (Supplementary Fig. 2c). Cell type proportions are known determinants of blood transcriptome profiles. Thus, we next further explored the genes whose expression levels were strongly associated with differential blood cell counts. This set included known marker genes such as *CD96* for lymphocytes^40^ and *SIGLEC8* for eosinophils^41^ (Fig. 1f and Supplementary Fig. 2d). We then computed cell-type specificity scores for each gene using publicly available scRNA-seq data from healthy peripheral blood^25,42^ (see “Methods”, Supplementary Data 2). Our analysis revealed that genes with high specificity scores for a given cell type were significantly enriched among those whose expression variability was predominantly attributable to the relative abundance of the same cell type (Fig. 1f and Supplementary Fig. 2e). By integrating cell-type specificity with variance decomposition in bulk data, we provide robust marker gene signatures for four major leukocyte populations—monocytes, eosinophils, neutrophils, and lymphocytes, which may be used to enhance the accuracy of cellular deconvolution in bulk data analyses.

Donor-specific expression variation was observed in numerous genes, notable examples included genes within the MHC locus, such as *HLA-DQB2* and *HLA-DQA2*, the *CD240D* (Rh blood group D antigen) and cell surface glycoprotein *MDGA1* (Fig. 1g and Supplementary Fig. 2f). Individual-specific gene signatures were followed up in subsequent analyses.

### Intra-individual gene expression variability surpasses inter-individual differences

**Fig. 2:**
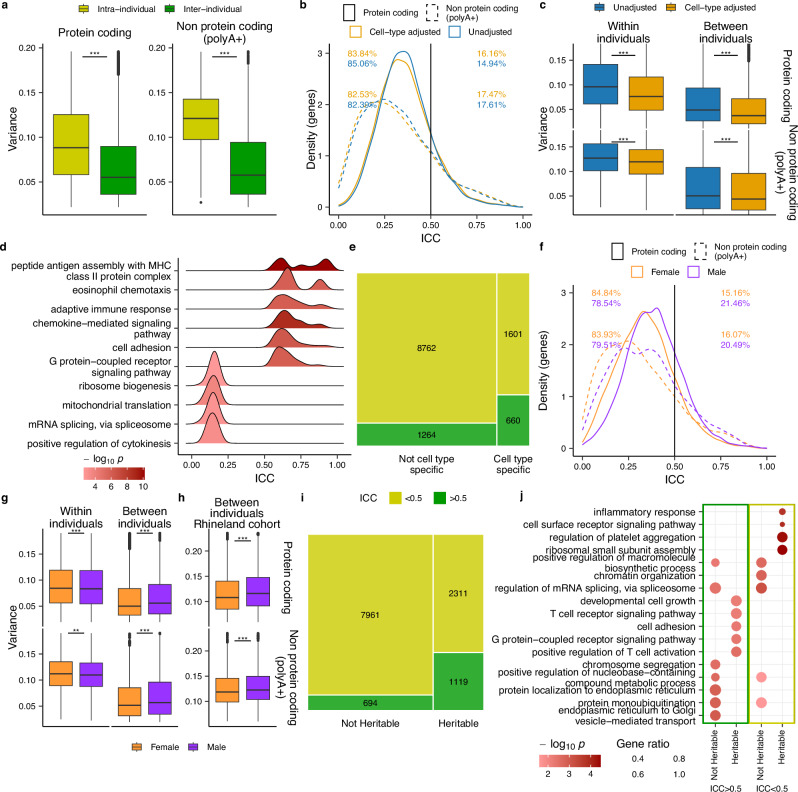
Intra- and inter-individual variation in gene expression. **a** Comparison of intra- and inter-individual variance for protein-coding (*n* = 11,641) and non-protein-coding (polyA^+^) (*n* = 2061) genes. Each data point represents a gene. Boxplots show median (centre line), interquartile range (IQR; box), and whiskers extend to 1.5 × IQR. Points represent outliers beyond the whiskers. Paired Mann–Whitney *U* test (two-sided) was used, and *p*-values were adjusted for multiple tests using Benjamini–Hochberg false discovery rate (FDR); *FDR < 0.05, **FDR < 0.01, ***FDR < 0.0001. **b** Density distributions of intraclass correlation coefficients (ICC) for protein-coding and non-protein-coding (polyA^+^) genes using cell-type adjusted (yellow) and unadjusted (blue) models. Each observation corresponds to a gene. **c** Comparison of intra- and inter-individual variance of protein-coding (*n* = 11,641) and non-protein-codi*n*g (polyA^+^) (*n* = 2061) genes estimated using cell-type adjusted (yellow) and unadjusted (blue) models. Boxplots are defined as in (**a**). Paired Mann–Whitney *U* test (two-sided), FDR-adjusted *p*-values, *FDR < 0.05, **FDR < 0.01, ***FDR < 0.0001. **d** Ridge plots of ICC values for the top and bottom 1000 genes enriched for selected GO terms. Colour intensity reflects significance of enrichment calculated using Fisher exact test (two-sided). **e** Mosaic plot showing distribution of genes with ICC < 0.5 (yellow) and ICC > 0.5 (green) relative to cell type marker status (see “Methods”). **f** Distribution of ICC values in protein-coding (*n* = 12,043) and non-protein-coding (polyA^+^) (*n* = 2,103) genes in females (orange) and males (purple). Each observation corresponds to a gene. **g** Comparison of intra- and inter-individual variance of protein-coding (*n* = 12,043) and non-protein-coding (polyA^+^) (*n* = 2103) genes in females and males. Boxplots are defined as in (**a**). Paired Mann–Whit*n*ey *U* test (two-sided), FDR-adjusted *p*-values, *FDR < 0.05, **FDR < 0.01, ***FDR < 0.0001. **h** Comparison of inter-individual variance of protein-coding (*n* = 10,264) and non-protein-coding (polyA^+^) (*n* = 888) genes in females and males in the validation cohort. Boxplots are defined as in (**a**). Paired Mann–Whitney *U* test (two-sided), FDR-adjusted *p*-values, *FDR < 0.05, **FDR < 0.01, ***FDR < 0.0001. **i** Mosaic plot showing distribution of genes with ICC < 0.5 (yellow) and ICC > 0.5 (green) against gene heritability. **j** GO terms enriched in genes sets classified based on ICC and heritability. Dot size is proportional to the gene ratio and colour denotes *p*-value of enrichment calculated using Fisher exact test (two-sided). Source data are provided as a Source Data file.

We next compared the variance in gene expression within samples from the same individuals, i.e., temporal variation, to that between individuals using intraclass correlation analysis (ICC) (Supplementary Data 2). Interestingly, the overall intra-individual variance in gene expression was higher than the inter-individual variance for both protein-coding (Paired Mann–Whitney *U* test, *p* < 10^−15^) and polyadenylated non-protein-coding (as annotated by GENCODE^43^ and captured by oligo(dT) selection, Paired Mann–Whitney *U* test, *p* < 10^−15^) genes (Fig. 2a and Supplementary Fig. 3a). About 85% of the protein-coding genes and 82% of polyadenylated non-protein-coding genes exhibited more variation within individuals than between individuals (ICC < 0.5, Fig. 2b). Although we noted a weak positive correlation between ICC and gene expression level (Spearman’s Rho = 0.31, *p* < 10^−15^), intra-individual variation was higher than inter-individual variation across different levels of expression (Supplementary Fig. 3b). When comparing gene expression variability to cell type composition variability assessed by routine laboratory analyses, we observed that most cell types exhibited less variation within individuals than between individuals. An exception was neutrophils, where approximately 55% of the variance originated from within individuals (Supplementary Fig. 3c). To further test the effect of cell type composition on inter- and intra-individual variation, we performed ICC analysis after adjusting for cell type composition, including 22 major leukocyte compositions, deconvoluted from bulk RNA-seq data using CIBERSORTx^44^ (Supplementary Data 2). While both inter- and intra-individual variance decreased significantly after adjusting for cell type composition (Paired Mann–Whitney *U* test, all, *p* < 10^−15^; Fig. 2c), the overall ICC distribution changed only marginally (Fig. 2b), and adjusted and unadjusted ICCs were highly correlated (Spearman’s Rho = 0.97, *p* < 10^−15^; Supplementary Fig. 3d). This indicates that cell type composition influences both variance components similarly and does not explain the predominance of intra-individual transcriptional variability. To ensure that these results were not driven by underlying health conditions and time of sampling, we repeated the analysis using either participants who did not self-report any acute or ongoing conditions (108 individuals, 313 samples) or samples that were collected only in the morning between 6 and 10 AM (554 samples from 219 individuals). Interestingly, both analyses confirmed the previous finding, with similar gene-level ICC values across the subsets (Spearman’s Rho = 0.94 and 0.97, *p* < 10^−15^; Supplementary Fig. 3e).

Genes with very low ICC values (i.e., high variation within individuals) included several ribosomal proteins and genes related to splicing and translation (Fig. 2d and Supplementary Fig. 3a and f). In contrast, a set of 2102 genes (15.3%), including genes from the HLA locus, T cell receptor-associated genes and genes involved in cell adhesion, chemokine-mediated signaling pathway and other immune-related genes such as *TMEM176A*, *TMEM176B*, *DEFA3*, and *C4BPA* were highly stable within individuals but varied considerably between individuals (Fig. 2d and Supplementary Fig. 3a and g). This group of genes with high inter-individual variance was enriched for cell-type specific genes (*χ*^2^ test of independence, *p* < 10^−15^), mostly linked to T-, B-, and NK cells (Fig. 2e and Supplementary Fig. 4a and b). Cell-type-specific genes were mostly involved in immune cell activation and migration processes (Supplementary Fig. 4c). This analysis demonstrates a strong link between gene function and expression variability patterns. Housekeeping genes exhibit high temporal variation with consistent expression across individuals, whereas each person retains a unique, time-stable gene signature. This individuality is closely associated with immune processes, particularly T and B cell immunity.

We conducted ICC analysis separately by sex on age-adjusted expression counts to identify genes and processes with distinct variation patterns between males and females (Supplementary Data 2). As expected, the global pattern of higher intra-individual variance than inter-individual variance was still observable in both males and females (Fig. 2f). Despite high correlation in the ICC values of the individual genes (Spearman’s Rho = 0.74, *p* < 10^−15^), several genes including *ADCK1, KLK1, IFNLR1*, and *LRRC4*, exhibited sex-specific inverse inter-vs-intra-individual variation (Supplementary Fig. 5a). Females exhibited higher intra-individual variance for both protein-coding and non-coding (polyA^+^) genes (Both Paired Mann–Whitney *U* test, *q* < 10^−15^; Fig. 2g). Since hormonal fluctuations and pro-inflammatory states associated with menstruation could account for this effect, we performed the analysis separately for pre- and post-menopausal women. Post-menopausal females showed lower variance in polyadenylated non-coding genes (Paired Mann–Whitney *U* test, *q* < 10^−15^), while elevated variance persisted in protein-coding genes (Paired Mann–Whitney *U* test, *q* < 10^−15^), showing that higher intra-individual variation in females is only partly explained by reproductive state (Supplementary Fig. 5b and c). Interestingly, inter-individual variance was higher in males when compared to both post- and pre-menopausal females (Both Paired Mann–Whitney *U* test, *q* < 10^−15^; Fig. 2g and Supplementary Fig. 5b and c). We validated our findings on sex-specific inter-individual variation using gene expression data in an independent, cross-sectional population-based sample set (*n* = 3480) from the Rhineland study (https://www.rheinland-studie.de/en/; Supplementary Data 1). This analysis confirmed higher inter-individual variance among male compared to female participants, a pattern that persisted even after stratifying women by menopausal status (Paired Mann–Whitney *U* test, all, *q* < 10^−15^; Fig. 2h and Supplementary Fig. 5d). Genes exhibiting greater variability within female individuals were enriched for processes related to blood circulation and immune pathways, including interleukin-6 and 17 signaling (Supplementary Fig. 5e and f). While sex-specific differences in gene expression are well known, our findings show that even the variability in gene expression differs between sexes, with a more stable signature of individuality in males and higher intra-individual variation in females, which is at least partially independent of their reproductive status.

### Heritable genes exhibit higher inter-individual expression variation in human blood

The variation in the expression of individual genes is influenced by environmental as well as genetic determinants. Twin studies have been used to quantify the genetic contribution to gene expression within a population using the heritability estimate (*h*^2^)^36,45^. To explore the impact of gene heritability on inter- and intra-individual expression variability, we utilized the heritability scores from the UK MultiMuTHER study, consisting of whole blood samples from 148 monozygotic and 166 dizygotic twins and 21 singletons from the TwinsUK study (see “Methods”, Supplementary Data 2). We compared the heritability scores calculated for each gene to the inter- and intra-individual variance measures. Genes exhibiting higher inter-individual variance (ICC > 0.5) showed markedly higher heritability compared to those with higher intra-individual variance (ICC < 0.5) (Mann–Whitney *U* test, *p* < 10^−15^; Supplementary Fig. 5g). Examining the number of genes with higher inter-individual or higher intra-individual variance against heritable (*h*^2^ lower CI > 0) and non-heritable (*h*^2^ lower CI ≤ 0) genes revealed a significant over-representation of heritable genes in the high inter-individual variance category (*χ*^2^ test of independence, *p* < 10^−15^; Fig. 2i). Heritable genes with high inter-individual variance were enriched in biological processes related to T cell activation and cell adhesion, among others, while notable processes with high intra-individual variation included platelet aggregation and ribosomal subunit assembly (Fig. 2j). This analysis shows that genes displaying higher variation between individuals are also more likely to be heritable, which is in line with genetic variation controlling “steady state” gene expression as a quantitative trait. Interestingly, we also identified genes, whose expression levels are heritable but still exhibit higher variation within individuals, suggesting contributions from stochastic processes or from biological and environmental factors that interact with genetic effects.

### Splicing variation dominates temporal transcriptional variability in peripheral blood

**Fig. 3:**
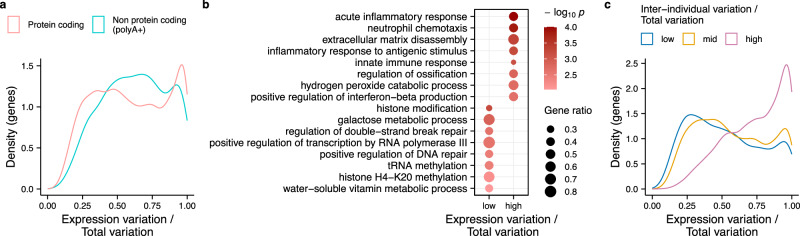
Variation in transcript usage. **a** Density distributions of the proportion of total transcriptional variability explained by expression level variation as opposed to splicing in protein-coding (*n* = 10,038) and non-protein-coding (polyA^+^) (*n* = 728) genes. Each observation corresponds to a gene. **b** GO terms enriched among the top and bottom 1000 protein-coding genes based on the relative contribution of expression level variation to total variability. Dot size is proportional to the gene ratio and colour corresponds to the *p*-value of enrichment calculated using Fisher exact test (two-sided). **c** Density plots showing the contribution of expression level variation to the total variability of genes with low (*n* = 2692), medium (*n* = 5382), and high (*n* = 2692) contribution of inter-individual variation to total variation. Each observation corresponds to a gene. Source data are provided as a Source Data file.

A gene can be expressed in several alternative transcript isoforms that can have different structures as well as functions^33,46^. Variation in the relative expression of isoforms within a given gene (splice ratios) across individuals and time thus represents a prominent element of overall transcriptional variability. To assess the extent of this source of transcriptional variation, we estimated the relative contribution of (a) splicing variation versus (b) expression level variation to the total variability in transcript abundance using an established deconvolution method^46^ (Supplementary Data 2). Differences in splicing patterns accounted for more than half of the transcriptional variation in 40% of protein-coding genes, which were enriched in cellular and metabolic processes and 31% of polyadenylated non-protein-coding genes consistently across different gene expression levels (Fig. 3a and b and Supplementary Fig. 6a). On the other hand, in about 17.9% of protein-coding genes, mainly involved in immune processes, and 16.7% of polyadenylated non-protein-coding genes, nearly all variation (>90%) originated from expression levels (Fig. 3a and b).

Linking this model for each gene to the relative contribution of intra- vs. inter-individual variation (see “Methods”^33^), we observed expression variation as the predominant source of inter-individual variation whereas splicing alterations contributed more to variability within individuals (Fig. 3c and Supplementary Fig. 6b). Moreover, intra-individual variance in transcript expression and splice ratios was significantly higher than inter-individual variance in both protein-coding and polyadenylated non-protein-coding genes (Paired Mann–Whitney *U* test, all, *q* < 10^−15^; Supplementary Fig. 6c). Stratifying for the absence of ongoing medical conditions and time of sampling (morning samples 6–10 AM) did not significantly change the results (Spearman’s Rho = 0.92 and 0.96, *p* < 10^−15^; Supplementary Fig. 6d). Taken together, we here show that across both protein-coding and polyadenylated non-protein-coding genes, splicing variation significantly contributes to the temporal transcriptional variation in peripheral blood samples.

### Seasonal variations significantly influence gene expression and transcript usage

**Fig. 4:**
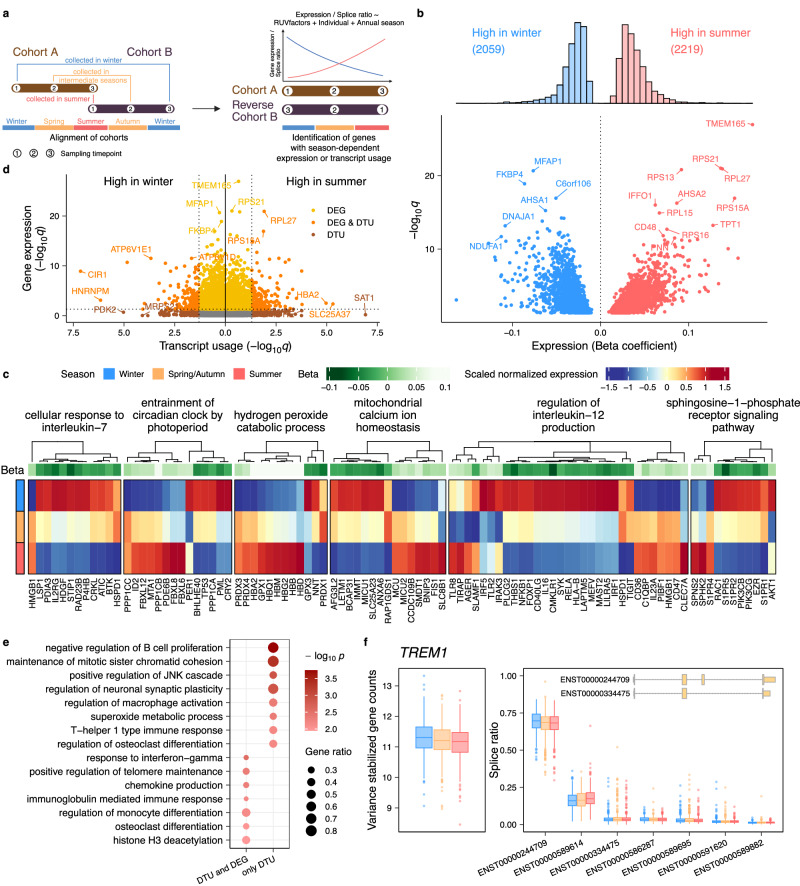
Seasonal variation in gene expression. **a** Schematic workflow. Created in BioRender. Kimmig, F. (2026) https://BioRender.com/7ry3hjq. **b** Volcano plot depicting effect size and FDR-adjusted *p*-values of differentially expressed genes between summer (pink) and winter (blue). Marginal histograms depict effect size distributions. Differential gene expression was assessed using linear mixed-effects models (two-sided) with Benjamini–Hochberg correction. Top significant genes are labelled. **c** Heatmap of seasonally differentially expressed genes enriched for selected biological processes. Average row-wise *z*-scores of normalized counts are plotted. Each column represents the mean expression profile of samples (biological replicate from individual donors) collected in a given season. **d** Scatter plot comparing significance of differential expression and differential transcript usage of DEGs and/or DTU genes. **e** GO terms enriched in genes with only differential transcript usage and genes with both differential gene expression and transcript usage across summer and winter. Dot size is proportional to the gene ratio and colour denotes enrichment *p*-value calculated using Fisher exact test (two-sided). **f** Seasonal changes in gene expression and splice ratio of *TREM1*. Box plots show median (centre line), interquartile range (IQR; box), and whiskers extend to 1.5 × IQR of variance stabilized expression or splice ratios. Points represent outliers beyond the whiskers. Each observation represents a sample (biological replicate from individual donors) collected in winter (*n* = 321), spring/autumn (*n* = 332), and summer (*n* = 317). Source data are provided as a Source Data file.

We next studied the systematic effect of annual seasons on gene expression and transcript usage. Samples in cohort A, collected between February and August, represent winter to summer transitions in northern Europe, while cohort B, collected between August and February, reflects summer to winter transition. To merge the two cohorts, we aligned winter (January–March; *n* = 321) and summer samples (July–September; *n* = 317) from both cohorts, treating spring (April–May; *n* = 265) and autumn (October–November; *n* = 67) samples as intermediate season (Fig. 4a). We applied linear mixed models (LMM) to identify the genes with seasonal expression and transcript usage (Fig. 4a and Supplementary Data 2).

We identified a total of 4278 genes that were differentially expressed (DEGs) across seasons (LMM, *q* < 0.05; Fig. 4b). Out of these, 2059 genes showed higher expression in winter while 2219 were upregulated in summer (Fig. 4b). Genes related to biological processes such as IL-12 production, response to IL-7, sphingosine-1 phosphate receptor signaling, and chromatin remodeling were upregulated in winter while several circadian rhythm genes and genes involved in Notch signaling were upregulated in summer (Fig. 4c and Supplementary Fig. 7a and b). We validated this analysis using the data of 3480 participants from the Rhineland study, which confirmed the involvement of seasonal DEGs in IL-7 response, sphingosine-1 phosphate receptor signaling and the circadian rhythm among other processes (Supplementary Fig. 7c and Supplementary Data 1). In contrast to the high number of DEGs between the seasons, only 722 genes showed differential transcript usage (DTU) between summer and winter (LMM, *q* < 0.05), out of which 351 genes showed both differential expression and transcript usage (Fig. 4d and Supplementary Fig. 7d). DTU genes were mainly enriched in immune processes such as response to interferon-gamma and Th1 response (Fig. 4e). For instance, *TREM1*, a cell surface receptor expressed by granulocytes and monocytes, exists in two primary isoforms: a membrane-bound form (*ENST00000244709*) and a soluble form (*ENST00000334475*). While the membrane receptor acts synergistically with the TLRs to activate proinflammatory cytokines, the soluble form acts as a counterbalancing principle^47^. We observed higher expression of *TREM1* in winter compared to summer (LMM, *q* = 0.01) along with subtle alterations in splice ratios (LMM, *q* = 0.047) favoring the membrane-bound isoform slightly more during winter (Fig. 4f). Analysis of individuals without ongoing self-reported medical conditions as well as only using morning samples generated similar results (Supplementary Fig. 7e). In summary, we observed intra-individual seasonal variations in gene and transcript expression, likely reflecting systematic annual shifts of environmental factors such as allergen and pathogen exposure, as well as fluctuations in daylight and diet. The associated gene sets can inform biomarker strategies that account for seasonal variations in gene expression, enhancing the accuracy of diagnostics and predictive models.

### Associations between gene co-expression modules, clinical parameters, and common medical conditions

**Fig. 5:**
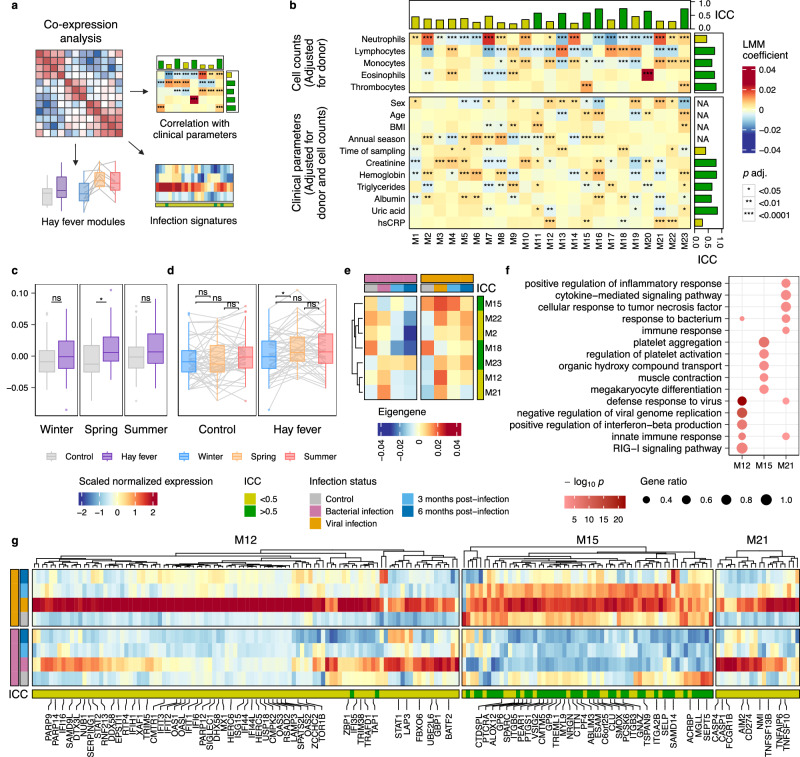
Module co-expression analysis. **a** Schematic representation of the workflow. Created in BioRender. Kimmig, F. (2026) https://BioRender.com/7ry3hjq. **b** Heatmap depicting associations between module eigengenes (columns) and clinical parameters (rows) estimated using linear mixed-effects models (two-sided). Models were adjusted for donor (random effects) and, where indicated, for donor and cell counts. *P*-values are corrected for multiple tests using Benjamini–Hochberg false discovery rate (FDR); *FDR < 0.05, ** FDR < 0.01, *** FDR < 0.0001. **c**, **d** Eigengene values of module M20 in individuals with hay fever and matched controls in cohort A (hay fever and control each: Winter, *n* = 44; Spring, *n* = 45; Summer, *n* = 43) across seasons (**c**) and pooled across seasons (**d**). Box plots show median (centre line), interquartile range (IQR; box), and whiskers extend to 1.5 × IQR. Points represent outliers beyond the whiskers. Between-group comparisons were performed using Mann–Whitney *U* test (two-sided) and paired comparisons using paired Mann–Whitney *U* test (two-sided), with FDR correction; *FDR < 0.05, **FDR < 0.01, ***FDR < 0.0001. **e** Heatmap showing average eigengene values of modules associated with infection across infection groups. Values represent averages across biological samples. **f** GO terms enriched in modules M12, M15, and M21. Dot size is proportional to the gene ratio and colour corresponds to the *p*-value of enrichment calculated using Fisher exact test (two-sided). **g** Heatmap of genes in modules M12, M15, and M21. Average row-wise *z*-scores of the normalized counts are plotted. Values represent averages across biological samples. Genes with module-membership score ≥ 0.8 are labelled. Source data are provided as a Source Data file.

Leveraging the dense clinical phenotype information of the longitudinal cohort, we next explored the gene expression patterns associated with different clinical parameters (e.g., lab tests) and frequent medical conditions such as allergies and common infections. We applied co-expression analysis to identify modules of genes with coordinated expression across individuals. This approach enables the detection of gene networks potentially linked to specific physiological or pathological states^11,48–52^. We identified 23 co-expression modules (M1–M23) from all protein-coding genes and assessed their association with different clinical parameters and phenotypes using linear mixed-effects models (Fig. 5a and b, Supplementary Fig. 8a and d, and Supplementary Data 2 and 3; see “Methods”). Module M16, linked to immune signaling and cytotoxic effector pathways, was associated with both sex and hemoglobin concentration (LMM, *q* < 0.05; Fig. 5b and Supplementary Fig. 8a–c). In addition, several modules were quantitatively associated with specific blood parameters. These included M4 and M10, carrying features of mitochondrial translation and tissue remodeling, respectively, which were associated with creatinine^53,54^, M9, also enriched for mitochondrial respiration^55^, with triglyceride levels, and M15 with thrombocytes (LMM, *q* < 0.05; Fig. 5b and Supplementary Fig. 8a–c). Module M20 highly correlated with the eosinophil content in the blood and contained several type II immunity marker genes, e.g., *IL5RA, ADORA3*, and *CEBPE* (Fig. 5b). Given the pivotal role of eosinophils in allergic responses, we analyzed the relationship between the expression of Module M20 and self-reported seasonal allergic rhinitis (hay fever) among participants. The module eigengene, reflecting the consensus expression of the module, exhibited season-dependent elevation in participants who reported hay fever compared to those who did not, particularly showing significantly higher values during the spring season among hay fever individuals (Mann–Whitney *U* test, *q* = 0.018; Fig. 5c and d). This season-specific upregulation was further corroborated by the expression profiles of genes within Module M20, which also exhibited increased levels during spring (Supplementary Fig. 8e).

Next, we sought to identify modules with expression patterns associated with self-reported minor infectious events. Reported infections were categorized as likely bacterial (*n* = 18) or viral infections (*n* = 14) (see “Methods”), and samples collected during the acute phase were compared to those collected 3 or 6 months after the reported infection, as well as to age-, sex-, and season-matched control samples from individuals without reported acute infections matched specifically to the acute phase samples (Supplementary Fig. 8f and g). Several modules, including M2, M12, M15, M21, M22, and M23, exhibited altered expression of eigengenes during infections compared to controls (Fig. 5e and f and Supplementary Fig. 8h). Notably, distinct modules were identified for bacterial and viral infections, some with longitudinal expression patterns (Fig. 5e). M12, upregulated during acute viral immune episodes, was enriched in defense response genes associated with viruses and interferon-beta production, e.g., *RSAD2, IFI27, IFI6* (Fig. 5f and g). M15 displayed sustained upregulation not only during the acute viral infection but also up to 3 months post-infection (Fig. 5g) and was enriched for genes associated with megakaryocyte and platelet differentiation, similar to a previously identified module relevant in severe SARS-CoV2 infection (Fig. 5f)^11^. Likewise, module M23 retained an infection-associated signature for up to 6 months post-infection, suggesting that even minor immunological insults can have lasting effects on the blood transcriptome. In summary, we demonstrate robust set of associations between co-expression modules and distinct clinical parameters, highlighting their potential as sensitive biomarkers for immunological states and previous infections.

### Impact of intra-individual variability on the accuracy of cohort-based transcriptomic studies

**Fig. 6:**
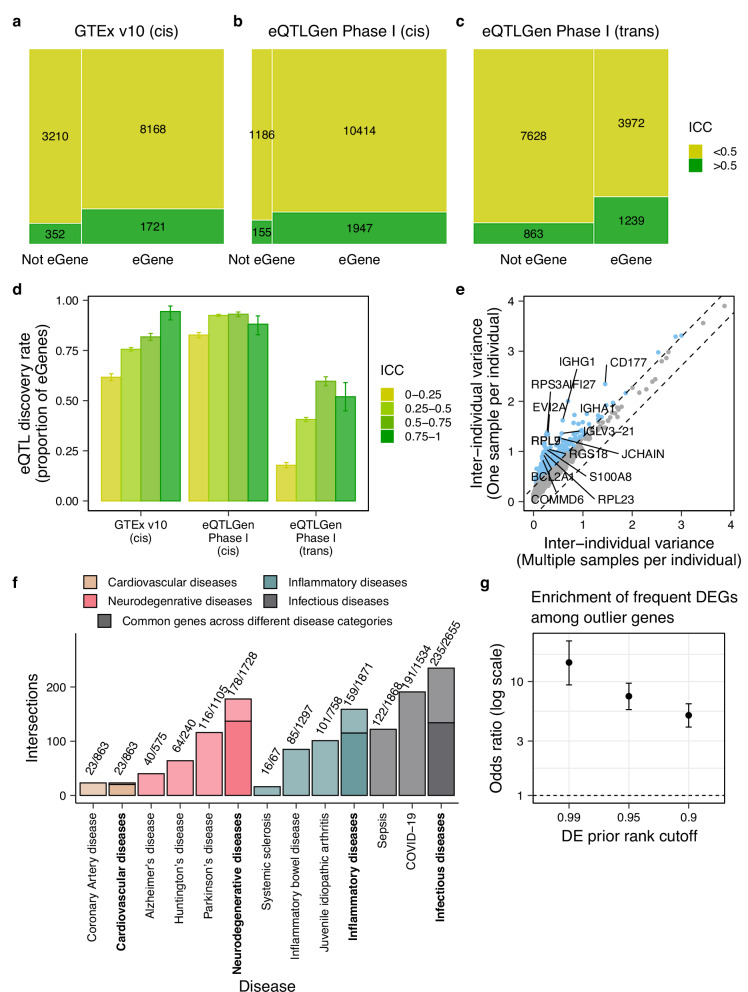
Impact of intra-individual variability on cohort-based research. **a–c** Mosaic plot showing distribution of genes with ICC < 0.5 (yellow) and ICC > 0.5 (green) relative to eGenes across different eQTL datasets. eGenes are defined as genes with at least one significant eQTL. **d** eQTL discovery rates across ICC bins in different eQTL datasets. Bars represent the proportion of eGenes within a given ICC bin. Error bars indicate 95% confidence intervals derived from a binomial test (two-sided). Each observation represents an aggregated proportion rather than individual gene. The number of genes contributing to each estimate ordered by increasing ICC bin are as follows: GTEx v10 (cis) (*n* = 3110; 8260; 1875; 198), eQTLGen Phase I (cis) (*n* = 3214; 8376; 1900; 202), eQTLGen Phase I (trans) (*n* = 3214; 8376; 1900; 202). **e** Comparison of inter-individual variance estimated from single versus multiple samples per individual (*n* = 13,702). Each observation represents a gene. Genes with differences in the two estimates greater than twice the standard deviation of the variance calculated using multiple samples per individual (outlier genes) are highlighted in blue and selected genes are labeled. **f** Number of overlapping outlier genes with differentially expressed genes identified in the transcriptomic analysis of different diseases. **g** Enrichment of “frequent DEGs” with different DE prior rank cutoffs among outlier genes. Points represent odds ratio and error bars correspond to 95% confidence intervals of the odds ratio estimated using Fisher exact test (two-sided). Number of outlier genes and frequent DEGs used to compute each odds ratio and provided in Supplementary Table 2. Source data are provided as a Source Data file.

We next evaluated the potential impact of high intra-individual variability on the reliability of transcriptomic analyses commonly used in clinical and population-based studies, including eQTL mapping, biomarker discovery, and differential gene expression (DGE). Using eQTLs identified from whole blood eQTL studies—cis-eQTLs from GTEx v10 ^56^and cis- and trans-eQTLs from eQTLGen Phase I^57^—we found that eGenes (genes with at least one eQTL) were significantly enriched among genes with higher inter-individual relative to intra-individual variance (all *χ*^2^ test of independence, *p* < 10^−15^; Fig. 6a–c and Supplementary Data 2). Accordingly, eQTL discovery rate and cross-cohort replication rates increased with ICC (Fig. 6d and Supplementary Fig. 9a), indicating that genes with stable expression over time are more likely to show reproducible genetic associations. While ICC showed only a weak relationship with eQTL effect size in GTEx, as measured by absolute slope (Spearman’s Rho = 0.12, *p* < 10^−12^), stronger associations were observed with eQTLGen z-scores (cis: Rho = 0.31; trans: Rho = 0.28, *p* < 10^−15^) and with the number of detected eQTLs per gene (GTEx: Rho = 0.17; eQTLGen cis: Rho = 0.24; trans: Rho = 0.34, *p* < 10^−15^; Supplementary Fig. 9b and c). Together, these results demonstrate that intra-individual expression variability constrains the detectability and reproducibility of eQTLs, while having little impact on the underlying magnitude of genetic effects.

Finally, to assess the effect of intra-individual variability on clinically relevant expression signatures, we compared inter-individual variance estimates from a single randomly selected sample per individual to those obtained using repeated sampling (Fig. 6e). Remarkably, we identified 508 genes exhibiting substantially higher (more than twice the standard deviation) inter-individual variance when calculated with only one sample per individual (hereafter named “outlier genes”; Supplementary Data 2), highlighting their sensitivity to sampling designs in cross-sectional study designs. The “outlier genes” were enriched in biological processes such as innate immune response, antimicrobial response, and oxygen transport (Supplementary Fig. 9d). These genes also had significantly lower eQTL discovery rates (*χ*^2^ test, *p* = 0.0006) and heritability compared to all other genes in the study (Mann–Whitney *U* test, *p* < 10^−15^; Supplementary Fig. 9e and f), suggesting that their elevated apparent inter-individual variability is driven by temporal instability rather than stable genetic regulation.

These outlier genes overlapped significantly with disease-associated DEGs reported across various conditions (Fisher exact test, *q* < 0.05 for 8 out of 9 diseases tested), including cardiovascular diseases (e.g., coronary artery disease^58^), neurodegenerative diseases (e.g., Alzheimer’s^21^, Parkinson’s^22^, and Huntington’s disease^23^), inflammatory conditions (e.g., inflammatory bowel disease^59,60^, juvenile idiopathic arthritis^60^, and systemic sclerosis^61^), and infectious diseases (e.g., COVID-19^11,14,27^ and sepsis^62^; Fig. 6f). Consistent with this observation, outlier genes were significantly enriched for previously described “frequent DEGs”^63^ across multiple prior rank thresholds (Fisher exact test, *q* < 0.05), which reflect how consistently genes have been identified as differentially expressed across independent studies (Fig. 6g, Supplementary Fig. 9g, and Supplementary Data 2). Notably, “frequent DEGs” and “outlier genes” were enriched for similar biological processes (Supplementary Fig. 9d), suggesting that pathways repeatedly detected across transcriptomic studies tend to show elevated temporal variability.

Together, these results show that intra-individual variability can inflate apparent inter-individual differences and reduce the reproducibility of genetic associations. Clinically, these results argue for biomarker selection informed by temporal gene-expression stability and against overinterpretation of bulk signals from single-timepoint sampling.

## Discussion

Peripheral blood is the most routinely available biosample in clinical practice^3,4^. Transcriptome analysis from peripheral blood has emerged as a tool in precision diagnostics, supported by more than a decade of studies from infection and inflammation^11–19^, cancer^8–10,64,65^, to neurodegeneration that have proposed numerous candidate mRNA biomarker signatures, e.g., for preclinical disease states. Prior longitudinal transcriptomic studies have provided important insights into gene expression dynamics, but primarily in cell-type-restricted or disease-focused settings^28,29^; however, surprisingly little is known about the physiological temporal variation of gene expression signatures from peripheral blood at population-level.

To build a healthy reference resource for guiding clinical interpretations of mRNA markers, we here set out to characterize fluctuations of blood transcriptomes in a large cohort of 333 individuals. Individuals were sampled three times longitudinally over the course of six months. The availability of validated phenotypes at every time point, ranging from comprehensive health questionnaires to thorough clinical routine lab tests, enabled the discernment of sources of variation within versus variation between individuals.

Strikingly, we identified substantial temporal variation of gene-level expression within individuals, which is by far exceeding inter-individual variation. We used expression heritability estimates from an independent UK twins cohort to assess the impact of genetic variation on gene-level expression. As anticipated, transcripts displaying heritable expression levels showed a relatively higher proportion of inter-individual variation. Nevertheless, most heritable genes still adhered to the trend of dominant temporal, or intra-individual variation. Cellular composition explained only a fraction of the observed variability. Although adjustment for measured and inferred cell-type proportions reduced variance, most genes still showed greater within-individual than between-individual variability. This excess intra-individual variability indicates that gene expression variation is shaped not only by stable, cell-intrinsic patterns but also by context-dependent, partially heritable responses. Identifying the biological and environmental contexts that shape these dynamics is a prerequisite for mechanistic interpretation. Further separating deterministic effects from potential stochastic noise will be critical for the reliable clinical use of transcriptomic biomarkers. The results align with previous findings from the 500FG project on SNP-dependent cytokine responses^66^, and are further supported by single-cell analyses demonstrating substantial plasticity in gene expression among circulating immune cells, even under physiological conditions^67–69^.

As we found that physiologically hypervariable transcripts are often contained in published disease mRNA biomarker sets, this has important clinical and methodological implications. First, high intra-individual heterogeneity limits the accuracy of single timepoint measurements, reducing the reliability of such markers. Genes with higher intra-individual variability also suffer from lower eQTL discovery and replication rates, consistent with previous observations that eQTL detection improves after correction for expression principal components^70^. Similar effects of temporal variability on eQTL detection were reported by Rondina et al. in platelets, although platelet expression was markedly more stable than expression in whole blood^28^. Together, these findings suggest that temporal instability diminishes the detectability of true biological associations, whether genetic or disease-related. Consequently, biomarker discovery and eQTL analysis cohorts may require larger than previously anticipated sample sizes to detect reliable effects. We flag outlier transcripts where inter-individual variation might be overestimated when intra-individual variation is ignored, rendering them more susceptible to spurious associations in cross-sectional settings. These outlier transcripts are significantly enriched for previously described “frequent DEGs”^63^, suggesting that recurrent different expression signals across studies may, in part, reflect underlying temporal variability rather than true trait associations. While genes and pathways recurrently detected across transcriptomic studies may capture shared pathophysiological mechanisms, our findings indicate that some of these signals may also arise from intrinsic physiological variability. Together, these observations underscore the importance of accounting for temporal variation when interpreting disease-associated signatures and imply that repeated measurements and combined marker sets, such as co-expression modules, will often be needed for diagnostic purposes. Second, we show that there are remarkable differences of intra-individual variation between biological sexes. Previous studies have demonstrated evolutionary conserved sexual dimorphism of mRNA levels in various organ systems^71–73^. We here extend this observation to sex-dependent intra- and inter-individual variation. In males, we show a shift towards higher inter-individual variation, while in females, intra-individual variation is higher compared to men. Biological terms linked to expression differences within male individuals were linked to energy homeostasis and metabolism, e.g., amino acid synthesis, autophagy, mitochondrion organization, whereas longitudinal variation in females was associated to immune-related features, e.g., neutrophil activation, IL6- and IL17-signalling. As some of the detected cytokines and their pathways have been previously shown to vary in blood with the menstrual cycle^74^, we assume that much of the female variation in gene expression might be linked to hormonal regulation and an increased pro-inflammatory tone during menstruation. Interestingly, females of post-menopausal status still exhibited a greater degree of intra-individual variability compared to males. This observation suggests that factors beyond hormonal fluctuation during the menstrual cycle may account for this phenomenon. The results highlight the importance of sex-sensitive design in clinical mRNA biomarker studies, including stratified analysis and reporting, to correctly capture biological variation in gene expression. Third, mRNA biomarker studies often neglect differential transcript usage (DTU) as a pivotal layer of functional complexity of the transcriptome. In a previous population-level study on cell lines from the 1000 Genomes project, we had shown that DTU was reflecting the genetic distance between populations, whereas within a population gene expression level variation prevails^33^. Strikingly, our results in peripheral blood indicate that changes in splicing patterns are significantly contributing to gene expression variation, particularly in genes that show high intra-individual variation, suggesting that differential transcript usage must be considered when studying transcriptomic changes in a human population. Finally, there was a substantial influence of annual seasons on gene-level expression as well as transcript usage. We observed higher expression of immune and interleukin signalling genes in winter, such as *TREM1*, which also showed slight shift in the usage of the pro-inflammatory isoform in winter. We also observed seasonal differences in the expression of circadian clock genes such as *ID2*, *PER1*, and *CRY2* as previously reported^75^. This seasonality of gene expression thus clearly mandates stratifying analysis for month of sampling in human cohort studies.

However, according to our data, although numerous genes exhibit significant temporal variation among individuals, each person maintains a consistent gene signature of individuality over time, particularly associated with various immune processes such as T and B cell immunity. The exact causes of this observation may be complex. On the one hand, this could be influenced by rare functional genetic variants as described in ref. ^76^; however, given the specificity of the signature for adaptive immunity-related cells and processes, this may also corroborate the hypothesis of fixed individual immune network states, which have been proposed in the context of vaccination studies^77^. Although their exact nature is still enigmatic, such temporally stable personal immune states are likely independent of disease condition or medication use^78^.

Several limitations of our study design should be considered. Our core study was conducted within a confined geographical region in Central Europe, which may limit the generalizability of our findings to populations with different genetic, environmental, or lifestyle factors. Second, our cohort size of 333 individuals provided sufficient statistical power for our analyses. However, while we validated several key findings in a larger population-representative sample, our results may not fully capture the heterogeneity of the general population. Third, our RNA sequencing approach excluded shorter fragments and relied on oligo(dT)-based selection of polyadenylated transcripts; consequently, our analysis does not capture the full transcriptomic landscape, including many long and short non-coding RNAs with potential regulatory functions, and is estimated to cover only ~20–40% of all non-coding transcripts^79,80^. Fourth, despite rigorous normalization and quality control, methodological challenges inherent to longitudinal transcriptomic analyses remain. Both RUVg normalization and WGCNA co-expression analysis rely on assumptions of sample independence and do not explicitly model within-subject correlation. While we verified that RUVg reduced technical variation without attenuating major biological signals and accounted for repeated measures in the downstream analysis of co-expression modules, future methods that explicitly incorporate longitudinal correlation structures during normalization and network construction may further refine such analyses. Finally, our longitudinal sampling design, while covering a one-year period, did not account for short-term temporal dynamics (e.g., diurnal) fluctuations or long-term variations. Despite these limitations, our study provides a strong foundation for RNA biomarker discovery and highlights avenues for future research to enhance the robustness and generalizability of our findings.

In conclusion, our study offers significant insights into the dynamic fluctuations of blood gene expression and transcript usage over time, emphasizing higher intra-individual variation compared to inter-individual variation across most genes, alongside notable sex-specific differences which are linked to specific immune pathways, including IL17/IL23 immunity. These intra-individual variations cannot be explained by cell type fluctuations alone, but we explain the influence of specific physiological sources of variation, such as seasonal changes, diurnal time of sampling, allergies, and self-limiting infections by mapping variation down to the most explanatory transcript sets.

Importantly, we also uncover stable differences between individuals, which we call signature of individuality. Genes related to that signature are highly specific to individual cell types (CD8, CD4 T- and B-Cells) and may reflect a temporally stable underlying immune network state. These findings have direct implications for the design and interpretation of transcriptomic studies in clinical cohorts. Genes with high inter-individual and low intra-individual variability should be prioritized for eQTL analyses (ICC threshold of ~0.5 for trans-eQTL analysis and single-cohort cis-eQTL analysis and ~0.25 for multi-cohort cis-eQTL analysis) and biomarker development, while test-retest designs and cross-cohort validation are essential to assess temporal robustness and generalizability. Moreover, sex, seasonal effects, and other major technical, environmental, and other biological sources of variations, such as time of blood draw should be explicitly modelled, and cell type composition should be carefully accounted for in blood RNA-seq analyses. Our study provides a quantitative framework and a well-characterized dataset for future investigations of gene regulation and mRNA biomarkers in peripheral blood.

## Methods

All research conducted in this study complied with relevant regulations. In particular, all research involving human participants was conducted in accordance with the Declaration of Helsinki. The study protocol regarding the longitudinal cohort (Cohort 1) was approved by the Ethics Committee Research of University Hospitals Leuven (Belgium). TwinsUK, which Cohort 2 is a subset of, main ethics was approved by the NHS London–London Bridge Research Ethics Committee and by Guy’s and St Thomas’ NHS Foundation Trust Research and Development (R&D). TwinsUK BioBank was approved by NHS North West-Liverpool East Research Ethics Committee. The Rhineland study (Cohort 3) received approval from the ethics committee of the Medical Faculty at the University of Bonn.

### Longitudinal study cohort

#### Ethics and consent

All procedures and protocols described in this study were approved by the Ethics Committee Research of University Hospitals Leuven (Belgium) (study ID S60030) and aligned with the Declaration of Helsinki and Belgian privacy laws. Participants were recruited from FGFP volunteers who expressed interest in participating in follow-up studies and the general population. Additional volunteers were recruited through online advertising. All participants were over 18 years old, resided in Belgium at the time of recruitment, and provided informed consent. A total of 500 participants were enrolled in this study and followed up for six months.

#### Metadata collection

Each month, participants were scheduled to visit their general practitioners. Blood samples and anthropometric characteristics (including length, weight, waist and hip circumference, blood pressure, and body temperature) were collected during these visits. Additionally, participants completed questionnaires about their overall health with their practitioners’ assistance, including information on their biological sex, diseases, allergies, menstrual cycle and infections during the study period.

#### Sample collection

Participants were instructed to avoid calorie intake for 8 h prior to blood sample withdrawal. Each blood sample consisted of a total volume of 20.5 ml, including 1 serum tube of 8 ml, 2 EDTA tubes of 4 ml, 1 fluoride tube of 2 ml, and 1 PAXgene Blood RNA tube of 2.5 ml. Blood analyses were conducted at The Centrum voor Medische Analyse (Herentals, Belgium). Seventy-three parameters were assessed, covering areas such as haematology, metabolism, inflammation, and the biochemistry of lipids, liver, and kidney (see Supplementary Data 1 for more details). One participant was excluded from the analysis due to diagnosis of cancer and ongoing treatment.

### RNA extraction and sequencing

RNA was isolated in QIAGEN’s QIAcube using the PAXgene Blood miRNA Kit from QIAGEN PreAnalytiX. RNA-sequencing libraries were prepared according to the Illumina TruSeq® messenger RNA (mRNA) sequencing protocol (TruSeq® RNA Seq Library Prep Kit v2) and sequenced on an Illumina NovaSeq 6000 (2 × 100 bp) with a median depth of 26 million reads.

### RNA-seq data analysis

The nf-core rnaseq pipeline (version 1.3) (https://github.com/nf-core/rnaseq)^81^ was used to pre-process the sequencing data. In brief, adapter and low-quality bases from the sequencing reads were trimmed using Trim Galore (0.5.0) (http://www.bioinformatics.babraham.ac.uk/projects/trim_galore/) and reads shorter than 35 bp after trimming were removed. The filtered reads were mapped to the human genome (GRCh38, GENCODE version 25) using STAR aligner (version 2.6.1d)^82^.

#### Gene expression QC

The expression counts of the genes were estimated using featureCounts (version 1.6.4)^83^. Genes with expression counts <5 in more than 25 percent of the samples were discarded and the remaining genes were normalized across samples using the DESeq2 median of ratios normalization method. All genes with biotype “protein_coding” as defined in GENCODE (v25)^43^ were classified as protein-coding. The set of polyadenylated non-protein-coding genes encompasses all genes not categorized as protein-coding captured by our library selection method. For dimensionality reduction analysis, gene expression was decomposed into between- and within-individual components using the function *withinVariation* from the mixOmics package (version 6.24.0^38^). Principal component analysis was then performed for inter- and intra-individual expression components separately using all genes that passed the previously mentioned expression cutoff (*n* = 14,166). Canonical correlations between the first six principal components and sample parameters such as sequencing run, cohort, annual season of sampling, age, sex, and BMI were calculated with the Bioconductor package variancePartition^84^.

#### Transcript expression QC

The transcript counts (TPM) were estimated using StringTie (version 1.3.5)^85^. TPM transcript expression levels were imported with the function *tximport* (“countsFromAbundance = dtuScaledTPM”) from the package *tximport* (version 1.28.0)^86^. In addition to the gene-level expression filter, transcripts expressed with ≤ 5 counts in more than 75 percent of the samples were also discarded (*DRIMSeq*, function *dmFilter*, version 1.28.0)^87^. The counts of the remaining transcripts were then normalized across samples with the DESeq2 normalization method. Splice ratios were calculated for all splice variants of a given gene in each sample as the proportion of transcript expression to gene expression, where the expression of the gene was defined as the sum over the counts of all splice variants of the gene.

### Cell-type deconvolution

For each bulk RNA-seq sample, relative immune cell-type proportions were estimated using CIBERSORTx^44^ with the LM22 reference matrix, which includes 22 major leukocyte populations such as naïve and memory B cells, plasma cells, CD8 + T cells, naïve and memory resting and activated CD4 + T cells, follicular helper T cells, regulatory T cells, γδ T cells, resting and activated NK cells, monocytes, macrophages (M0, M1, M2), resting and activated dendritic cells, resting and activated mast cells, eosinophils, and neutrophils.

### Intraclass correlation analysis

The intraclass correlation coefficient was computed for autosomal genes that passed the previously mentioned expression cutoff (*n* = 13,702) using the function *ICCest* from the R package ICC (version 2.4.0), which uses the between-group and within-group variance components from one-way ANOVA.

To account for variation in cell type composition, we estimated cell type-aware ICCs using linear mixed-effects models. To obtain orthogonal covariates that summarize the dominant axes of cell type variation while avoiding multicollinearity inherent to compositional proportions, principal component analysis was performed on log-transformed inferred cell type proportions and the first 5 principal components (PCs) were retained as covariates. For each gene, variance-stabilized expression values were modelled using a linear mixed-effects model with the following formula

1 $${{{\rm{expr}}}}_{{ij}}=\,{\beta }_{0}+\,{\sum }_{k}{\beta }_{k}.\,{{{\rm{cellPC}}}}_{{ijk}}+\,{u}_{i}+\,{\varepsilon }_{{ij}}$$

where ${{{\rm{expr}}}}_{{ij}}$ denotes the expression level of a given gene in individual $i$ at timepoint $j$, ${{{\rm{cellPC}}}}_{{ijk}}$ represents the $k$-th cell type PC for that sample, ${u}_{i}$ is a subject-specific random intercept capturing between-individual variability, and ${\varepsilon }_{{ij}}$ is the residual error capturing within-individual variability over time. Models were fitted using restricted maximum likelihood as implemented in the lme4 package in R (version 1.1–37). For each gene, variance components were extracted from the fitted model and cell type-adjusted ICC was defined as

2 $${{\rm{ICC}}}=\frac{{\sigma }_{{{\rm{between}}}}^{2}}{{\sigma }_{{{\rm{between}}}}^{2}+\,{\sigma }_{{{\rm{within}}}}^{2}}$$

where ${\sigma }_{{{\rm{between}}}}^{2}$ corresponds to the variance of the subject-specific random intercept and ${\sigma }_{{{\rm{within}}}}^{2}$ corresponds to the residual variance.

### Variance partition analysis

Quantitative partitioning of gene expression variance was performed on gene counts after variance stabilizing transformation (vst counts) using the Bioconductor package variancePartition (version 1.30.2)^84^. Variance attributable to individual, blood cell counts, sequencing run, cohort, time point of sampling, age, gender, BMI, and clinical parameters such as creatinine, hemoglobin, triglycerides and hsCRP was estimated using all samples without missing values in these variables (*n* = 738). All genes located on autosomes were considered in this analysis (*n* = 13,702).

### Removal of unwanted variation

To reduce the effect of the technical batches from cohort and sequencing run, we applied the factor analysis method RUVg (R package RUVSeq, version 1.28.0)^39^. This algorithm uses a list of negative control genes, i.e., genes that are known a priori to be similarly expressed in all samples, to detect hidden variables in expression data that capture unwanted variation. Including these factors in the analysis as confounders can reduce the effect of technical batches and has been shown to provide reasonable results even for repeated measures^39^. After excluding genes that lie on the sex chromosomes, twelve such synthetic factors were discovered from all samples (*n* = 970) using a set of house-keeping genes as negative controls. Two of these factors accounted for most of the technical batch variance and were included as covariates in the subsequent analyses.

### Cell type specificity

Specificity of gene expression regarding different blood cell types was measured with the tau index^42^. Briefly, a publicly available single-cell transcriptome dataset derived from fresh PBMCs and whole blood from healthy individuals^25^ was used to compute the tau score. The preprocessed Seurat object, including cell clusters, was adopted for the calculation. For each gene, the average expression in selected cell types was computed. For each of these cell types, the genes were partitioned into 10 equally sized groups based on the 10-quantiles of their average expression giving rise to an expression profile for each gene. Genes with a proportionate average expression <${10}^{-7}$ were not included in this division process and were assigned expression profile component 0. The tau index was defined as

3 $$\tau=\frac{{\sum }_{i=1}^{N}\left(1-{x}_{i}\right)}{N-1}$$

where *N* is the number of cell types and ${x}_{i}$ is the expression profile component normalized by the maximal component value. It was computed for each gene, yielding a score between 0 and 1, with 0 indicating low and 1 indicating high specificity (Supplementary Data 2). A gene is specific to the cell type or cell types where the expression profile component equals the maximum of all component values. Marker genes for a cell type were defined as the set of all genes specific only to this cell type with a cell type specificity score > 0.5 and a maximal component value ≥ 2.

The above-described computation was performed at two different resolutions regarding the selected cell types. A lower resolution considered eosinophils (*n* = 145), lymphocytes (*n* = 7757), monocytes (*n* = 2226), neutrophils (*n* = 7939), dendritic cells (*n* = 155), megakaryocytes (*n* = 399) and proliferative cells (*n* = 44) and the resulting specificity scores were used to test for enrichment of marker genes in the variance partition analysis. To compute specificity values at a higher resolution, cells were divided into CD4 + T cells (*n* = 4409), CD8 + T cells (*n* = 1209), B cells (*n* = 997), plasmablasts (*n* = 37), NK cells (*n* = 1105), dendritic cells (*n* = 155), eosinophils (*n* = 145), neutrophils (*n* = 7737), CD14 monocytes (*n* = 1897), CD16 monocytes (*n* = 329), megakaryocytes (*n* = 399), immature neutrophils (*n* = 202) and proliferative cells (*n* = 44). Scores derived from the higher resolution were used to characterize genes with high inter-individual variation identified in the ICC analysis.

### Enrichment in cell type markers

Marker genes for lymphocytes, monocytes, eosinophils and neutrophils were identified through specificity scores computed from a low resolution at cell type level as described in the “Method” section for cell type specificity. The enrichment of genes with high variance in expression explained by different cell type proportions in cell type markers was assessed using Fisher’s exact test. The test was performed for neutrophils, lymphocytes, monocytes and eosinophils on the top 1000 genes with the highest variance explained by the respective cell type (variance partition analysis) and all marker genes for that cell type as defined above.

### Sex segregated ICC analysis

Variance within and between individuals as well as the ICC (see method section on ICC analysis), was computed on age-adjusted residual variance stabilized counts for all genes located on autosomes or the X chromosome (14,146 genes) for female (141 individuals, 351 samples) and male (78 individuals, 203 samples) participants separately. Only samples collected in the morning between 6 and 10 AM from participants with two or more samples collected during this timeframe were included.

This analysis was repeated for men and pre- (90 individuals, 223 samples) and postmenopausal (36 individuals, 91 samples) women on non-adjusted variance-stabilized counts. Pregnant participants and women who reported a stop in their menstruation before the age of 40 were excluded. Characteristics of the participant subsets used in this analysis can be found in Supplementary Data 1.

### Heritability analysis

The heritability scores were derived from the MultiMuTHER study (a subset of TwinsUK), consisting of 335 European participants from the UK, including 148 monozygotic twins, 166 dizygotic twins and 21 singletons, with whole blood gene expression and metabolite levels measured at three clinical visits spanning a median of six years. TwinsUK main ethics was reviewed and approved by the NHS London—London Bridge Research Ethics Committee (REC reference EC/04/015) and by Guy’s and St Thomas’ NHS Foundation Trust Research and Development (R&D) in 2012. TwinsUK BioBank was approved by NHS North West—Liverpool East Research Ethics Committee (REC reference 19/NW/0187), IRAS ID 258513. All research therefore carried out in accordance with the ethical standards laid down in the 1964 Declaration.

Extracted RNA samples were prepared for sequencing using the Illumina TruSeq sample preparation kit, and samples were sequenced on an Illumina HiSeq 2000 machine according to the manufacturer’s instructions. 49 bp paired-end reads were aligned to the GRCh38 reference genome using STAR^82^ version 2.6.1a, using a quality score threshold of 255. The *quan* function from the R package QTLtools^88^ was used to calculated gene-level counts per million (CPMs). Genes were filtered to retain those with 5 or more CPMs in greater than or equal to 25% of samples. Trimmed mean of M-values between-sample normalization^89^ was then applied to gene-level CPMs to derive TMM-adjusted CPMs. A total of 16,292 genes were retained in downstream analyses.

We applied a rank-based inverse normal transformation to each gene individually using the *rntransform* function from the GenABEL package^90^. Gene expression levels were then adjusted for known RNA-Seq technical covariates using linear mixed-effects models, implemented using the *lmer* function from the lme4 R package. Sample median transcript integrity number^91^, median insert size, mean GC content, and RNA integrity number were included as fixed effects, and date of library preparation, date of RNA sequencing, and primer index included as random effects. Residuals from the above models were used to calculate gene expression heritability using the *twimlm* function from the mets package in *R* version 4.1.1. Genes with an *h*^2^ lower confidence interval above zero were considered heritable. Reported heritabilities are those calculated using the baseline visit for each study participant.

### Variability in gene expression versus variability in alternative splicing

For each gene (*n* = 10,766), total variation $\left({V}_{{{\rm{total}}}}\right)$ and variation from changes in expression levels $\left({V}_{{{\rm{expr}}}}\right)$ were calculated from transcript expression levels using a method developed by ref. ^46^. Inter- and intra-individual variation was computed for each gene by partitioning the total variation ${V}_{{{\rm{total}}}}$ into the variation within and between individuals applying the same reasoning that is used in ANOVA:

Let *k* be the number of individuals and ${m}_{j},1\le j\le k$, be the number of samples of individual *j*. Let further *g* be a gene with *t* alternative splice forms.

We denote the total number of samples as *N*, with ${e}_{{jl}}\left(i\right)$ the expression of splice form *i* in sample *l* of individual *j* and with ${\mu }_{i}=\frac{1}{N}{\sum }_{j=1}^{k}{\sum }_{l=1}^{{m}_{k}}{e}_{{jl}}\left(i\right)$ and ${\mu }_{{ij}}=\frac{1}{{m}_{j}}{\sum }_{l=1}^{{m}_{j}}{e}_{{jl}}\left(i\right)$ the mean expression of transcript *i* over all samples and the mean expression of transcript *i* in individual *j*, respectively. Then

4 $${V}_{{{\rm{total}}}} 	={\sum }_{i=1}^{t}{\sigma }_{i}={\sum }_{i=1}^{t}\frac{1}{N-1}{\sum }_{j=1}^{k}{\sum }_{l=1}^{{m}_{j}}{\left({e}_{{jl}}\left(i\right)-{\mu }_{i}\right)}^{2} \\ 	={\sum }_{i=1}^{t}\frac{1}{N-1}\left[{\sum }_{j=1}^{k}{\sum }_{l=1}^{{m}_{j}}{\left({e}_{{jl}}\left(i\right)-{\mu }_{{ij}}\right)}^{2}+{\sum }_{j=1}^{k}{m}_{j}{\left({\mu }_{{ij}}-{\mu }_{i}\right)}^{2}\right] \\ 	={V}_{{{\rm{within}}}}+{V}_{{{\rm{between}}}}$$

where ${\sigma }_{i}$ is the variance of the expression of splice form $i$.

Consequently, we can assess the proportion of variation in gene *g* that is introduced by individuals through $\frac{{V}_{{{\rm{between}}}}}{{V}_{{{\rm{total}}}}}$, where a value close to zero means that all the variation is intra-individual and a value close to one can be interpreted as all variation being introduced by differences between individuals. Analogously, we can divide ${V}_{{{\rm{expr}}}}$ into ${V}_{{{\rm{expr}}}\_{{\rm{within}}}}+\,{V}_{{{\rm{expr}}}\_{{\rm{between}}}}$ and determine the proportion of expression level variation in gene *g* that is introduced by individuals through $\frac{{V}_{{{\rm{expr}}}\_{{\rm{between}}}}}{{V}_{{{\rm{expr}}}}}{\scriptstyle{33}}$.

### Seasonal analysis

Differential gene expression and differential transcript usage between seasons were identified using LMM implemented in the Bioconductor package Maaslin2 (version 1.14.1)^92^. Samples collected in winter (January, February, March; *n* = 321) and summer (July, August, September; *n* = 317) between the two cohorts were aligned and the spring (April, May; *n* = 265) and autumn (October, November; *n* = 67) samples were used as the intermediate season samples. Seasons were then transformed into a numeric time scale with winter being 1, spring/autumn 2, and summer 3. LMM were constructed using individual and factors 2 (W_2) and 3 (W_3) from the RUV analysis as fixed effects and time as random effect. Only protein-coding genes were included in this analysis (*n* = 12,057).

### Functional enrichment analysis

All gene ontology enrichment analyses were conducted using the Bioconductor package topGO (version 2.46.0), with genes with similar expression level as the universe set. In the topGO analysis, the Fisher.elim *p*-value, calculated using the weight algorithm, of 0.05 was used as the significance threshold. For the ICC analysis, top 1000 genes with highest ICC and top 1000 genes with lowest ICC were used as test sets.

### Gene co-expression analysis

All protein-coding genes located on autosomes that met the previously mentioned expression threshold (*n* = 11,641) were used to create co-expression modules through the WGCNA package in *R*^48^. Pairwise gene correlations were calculated based on the variance-stabilized counts across all samples and a signed adjacency matrix was constructed by applying a soft threshold of 9. The Topology Overlap Matrix (TOM), derived from the adjacency matrix, was employed to construct a gene tree via hierarchical clustering. Subsequently, genes were grouped into modules based on the gene tree by using the function cutreeDynamic with the minimum module size of 15. Closely related modules were then merged using the function mergeCloseModules with parameter cutHeight set to 0.2. To link gene co-expression modules with clinical parameters and to visualize the expression profile within a module, the module eigengene values were computed for the samples. Module-trait associations were assessed through two types of linear mixed-effects models (LMMs).

5 $${{\rm{lmm}}}1\,={{\rm{eigengene}}} \sim {{\rm{trait}}}+\left(1| {{\rm{Individual}}}\_{{\rm{ID}}}\right)$$

was employed for all traits to adjust for donating individual while

6 $${{\rm{lmm}}}2={{\rm{eigengene}}} \sim {{\rm{neutrophils}}}+{{\rm{lymphocytes}}}+{{\rm{monocytes}}} \\+{{\rm{eosinophils}}}+{{\rm{thrombocytes}}}+{{\rm{trait}}}+\left(1|{{\rm{Individual}}}\_{{\rm{ID}}}\right)$$

was used on clinical parameters excluding cell counts to additionally account for cell type composition of the sample (nlme, function *lme*, version 3.1–162^93^). Significance of associations was determined by comparing the full to a reduced model. All variables were standardized prior to testing to ensure comparability of estimates.

#### Hay fever analysis

Fifty-nine individuals reported to suffer from hay fever. Out of these 8 individuals reported to also have asthma and were excluded from subsequent analyses. Individuals without hay fever, other seasonal allergies or asthma matched for sex, age (±12 years) and cohort were selected as controls for the remaining 51 individuals (Cohort A, *n* = 45; Cohort B, *n* = 6). To explore season-dependent changes in the molecular signatures underlying hay fever, we compared the eigengene of the eosinophil-related module M20 between participants with hay fever and the selected controls within winter, spring and summer and investigated the individual trajectories across annual seasons.

#### Infection analysis

Samples collected during the acute phase of an infection were divided into the three groups viral, bacterial, and other. Viral infections included upper airway infections (*n* = 3), common cold (*n* = 10), pharyngitis (*n* = 1) and herpes labialis (*n* = 4) while bronchitis/sinusitis (*n* = 7), tonsilitis (*n* = 2), lupus and rheumatoid arthritis related infections (*n* = 2), cystitis, dental infections, hordeolum, implant infections, mastitis and pertussis (each *n* = 1) were classified as bacterial infections. Due to the limited impact of local herpes reactivation on the blood transcriptome, samples with this type of infection were excluded from the subsequent analysis to increase power. Control samples were chosen based on sex, age and annual season from individuals who did not report an infection at or a month before any of the sampling timepoints or any sustained medical conditions to match individuals with acute infections. Infection-related modules were then identified using both functional enrichment analysis as well as module eigengene expression in acute infection timepoint samples compared with their matched controls. To visually investigate the longitudinal disease course of infections, we categorized all three samples obtained from individuals experiencing an infection during one of the sampling timepoints into three distinct groups: Bacterial/Viral infection (including all samples obtained when an infection was reported at the timepoint of sampling), 3 months post-infection (consisting of samples from individuals who had reported an infection at the preceding sampling timepoint and had since recovered), and 6 months post-infection (encompassing samples where the infection occurred two sampling timepoints in the past).

### eQTL-based analysis

To relate longitudinal expression stability to genetic regulation, we integrated our inter- and intra-individual variance estimates with large-scale blood eQTL resources. We used summary-level eQTL results from GTEx whole blood^56^ (v10) for cis-eQTL discovery and eQTLGen consortium (phase I^57^) for both cis- and trans-eQTLs in peripheral blood. Cis- and trans-eGenes were defined according to the consortiums’ reported gene-level significance. Gene identifiers were harmonized across datasets using Ensembl gene IDs and all analyses were restricted to the intersection of genes present in the longitudinal expression dataset and the respective eQTL resource.

Gene-level eQTL discovery rates were computed as the proportion of genes classified as eGenes within a given gene set or ICC bin. Regression slopes (GTEx) and *Z*-scores (eQTLGen) were used as proxies for effect size. To evaluate the reproducibility of genetic regulation as a function of ICC, we performed reciprocal replication analyses between GTEx and eQTLGen for cis-eQTLs. Replication rates were computed within ICC bins as the proportion of discovery eGenes that were also classified as eGenes in the replication dataset.

### Outlier genes and frequent differentially expressed gene (DEG) analysis

To quantify the impact of intra-individual variability on cross-sectional estimates of inter-individual differences, we compared variance estimates for autosomal genes that passed the previously mentioned expression cutoff (*n* = 13,702) derived from single timepoint sampling to those obtained using repeated measurements per individual. Inter-individual variance was calculated after randomly selecting one sample per individual and compared to variance estimates from the full repeated-measure dataset. Genes were classified as “outlier genes” if inter-individual variance estimated from single time-point sampling exceeded the repeated-measure estimate by more than two standard deviations of the genome-wide distribution. Outlier genes were used in downstream analyses to assess overlap with published disease-associated gene sets and frequent differentially expressed genes.

To assess whether genes with high intra-individual variability are enriched among recurrently reported differentially expressed genes, we compared our outlier gene set to a published catalog of “frequent DEGs”^63^. Crow et al. assigned a prior probability of differential expression to each gene using a Bayesian meta-analysis framework across over 600 transcriptomic studies. Frequent DEG sets were defined using prior probability thresholds of 0.90, 0.95, and 0.99, with higher thresholds indicating more consistently reported differential expression. Overlap between outlier genes and frequent DEGs was assessed using Fisher’s exact test, with the background defined as all genes included in the variance analysis. Enrichment analyses were performed separately for each prior probability threshold.

### Statistical analyses

All analyses were performed using R Statistical Software (version 4.3.1). All statistical tests used were two-sided. Correlations were assessed with the Spearman correlation method, unless specified otherwise. Correction for multiple testing was performed where applicable using the Benjamini and Hochberg method.

### Rhineland study cohort

To validate our results on sex-specific expression variation and seasonal expression patterns, data from an independent cohort was analyzed:

#### Study population, ethics, and consent

The Rhineland Study is an on-going population-based cohort study in Bonn, Germany. Invitations to join the study are extended to all residents of two geographically defined areas of Bonn who are 30 years of age or older and have sufficient German language proficiency to provide informed consent. The study received approval from the ethics committee of the Medical Faculty at the University of Bonn (reference ID: 338/15). It is conducted in compliance with the International Conference on Harmonization (ICH) Good Clinical Practice (GCP) standards (ICH-GCP). Written informed consent was obtained from all participants in accordance with the Declaration of Helsinki.

#### Metadata collection

Age and sex were collected through self-reports. Differential blood cell counts were measured at the Central Laboratory of the University Hospital Bonn using EDTA-whole blood samples analyzed on a Sysmex XN9000 hematological analyzer. Menopause status (yes/no) was assessed using self-reported information regarding current menstruation, surgical procedures in the uterus or ovaries, and the use of hormonal contraception.

#### RNA extraction and sequencing

Total RNA was isolated from 3480 peripheral blood samples collected in PAXgene Blood RNA tubes (PreAnalytix/Qiagen), following the manufacturer’s protocol. Total RNA extraction was performed using the PAXgene Blood miRNA Kit, adhering to the manufacturer’s guidelines. RNA integrity and quantity were evaluated using the TapeStation RNA assay on a TapeStation 4200 instrument (Agilent). For library preparation, 750 ng of total RNA was used to generate next-generation sequencing (NGS) libraries with the TruSeq Stranded Total RNA Kit (Illumina), incorporating Ribo-Zero Globin reduction, per the manufacturer’s instructions. Library size distribution was assessed with the D1000 TapeStation assay on the TapeStation 4200, and library quantification was carried out using the Qubit HS dsDNA assay (Invitrogen). Libraries were clustered at a final concentration of 250 pM on a NovaSeq 6000 instrument. For the first batch of 3000 samples, NovaSeq S2 v1 chemistry was used in XP mode, while NovaSeq S4 v1.5 chemistry was applied to the final batch of 480 samples. Paired-end sequencing (2 × 50 cycles) was conducted, followed by demultiplexing using bcl2fastq2 v2.20. Sequencing data quality control was performed using FastQC (v0.11.9).

#### RNA-seq analysis

Gene expression levels from the RNA-Seq data were obtained by aligning sequencing reads to the GRCh38.p13 human reference genome (Ensembl) using STAR v2.7.1. The count matrix was generated with the STAR –quantMode GeneCounts command, using the GRCh38.101 human gene annotation. Genes included in the analysis were those expressed in at least 5% of participants and with an average expression greater than 15 reads.

#### Validation of sex-specific gene expression variation

Study participants with complete data on gene expression, age and menopause status were considered for this analysis and anthropometric data for these individuals stratified by sex and menopausal status is included in Supplementary Data 1. Due to the availability of RNA sequencing data at only one timepoint per individual in the Rhineland cohort, only sex-specific variation between individuals was analyzed. Gene expression variance as a proxy for inter-individual variation was computed on age-adjusted residual variance stabilized counts for male (*n* = 1335) and female (*n* = 1607) participants as well as on non-adjusted variance stabilized counts for men and pre- (*n* = 568) and post-menopausal (*n* = 928) women separately. Only genes located on autosomes and on the X chromosome were considered in this analysis (11,152 genes).

#### Validation of seasonal analysis

To validate differential gene expression between seasons, samples from the Rhineland cohort were grouped according to their date of sampling into samples collected in winter (*n* = 1007), spring/autumn (*n* = 895, 332/895 in spring, 563/895 in autumn) and summer (*n* = 887). Only participants with complete data on gene expression, age, sex, and blood cell counts were included in this analysis (Supplementary Data 1). Linear mixed effect models were constructed as described above for all protein-coding genes (*n* = 10,161), with age and sex as additional covariates. Genes with replicated differential expression and directionality of expression were further characterized through functional enrichment analysis.

### Reporting summary

Further information on research design is available in the Nature Portfolio Reporting Summary linked to this article.

## Supplementary information

Supplementary Information

Description of Additional Supplementary Files

Supplementary Data 1

Supplementary Data 2

Supplementary Data 3

Reporting Summary

Figure 1 source data

Figure 2 source data

Figure 3 source data

Figure 4 source data

Figure 5 source data

Figure 6 source data

Figure S2 source data

Figure S3 source data

Figure S4 source data

Figure S5 source data

Figure S6 source data

Figure S7 source data

Figure S8 source data

Figure S9 source data

Transparent Peer Review file

## Source data

Source data

## Data Availability

RNA-seq data generated for Cohort 1 (333 individuals) have been deposited in the European Genome-Phenome Archive with accession code EGAD50000000859. To protect the privacy of the individuals, these datasets, including processed files and metadata are available under restricted access. Requests for access can be submitted directly in EGA. These requests are reviewed by the VIB Data Access Committee within a reasonable timeframe. Data can be shared for research purposes that are compatible with the original goal for which the data were collected. Prior to the sharing of any data, a Data Sharing Agreement with VIB must be completed that will include the necessary conditions to guarantee the protection of personal data and limit secondary use to a specified research activity. All data relating to TwinsUK samples, which Cohort 2 is a subset of, have been deposited to the TwinsUK BioResource data management team. These data are available by application to the Twin Research Executive Access Committee (TREC) at King’s College London. The TwinsUK BioResource is managed by TREC, which provides governance of access to TwinsUK data and samples to researchers investigating health, well-being or disease. TwinsUK data users are bound by data sharing agreement set out in the data access application form, which includes responsibilities with respect to third-party data sharing and maintaining participant privacy. Further responsibilities include a responsibility to acknowledge data sharing. Please see https://twinsuk.ac.uk/researchers/access-data-and-samples/request-access/ for additional information and to access data access proposal forms. The data from the Rhineland Study (Cohort 3) used in this manuscript are not publicly available due to data protection and privacy regulations. For the Rhineland Study, access can be obtained by submitting a formal request to the Data Access Committee (RS-DUAC) for academic, non-commercial research purposes, and in accordance with the Rhineland Study’s Data Use and Access Policy. The committee will review all requests within 4 weeks. Access may be subjected to a completed data transfer agreement. Requests for further information or to access the Rhineland Study’s dataset should be directed to RS-DUAC@dzne.de. Source data are provided with this paper.

## Code availability

The custom codes used in this study are available at GitHub (https://github.com/Systems-Immunology-IKMB/Temporal_gene_expression_variation)^94^.

## References

[1] Wang, Z., Gerstein, M. & Snyder, M. RNA-seq: a revolutionary tool for transcriptomics. *Nat. Rev. Genet.* **10**, 57–63 (2009). 19015660 10.1038/nrg2484 PMC2949280

[2] Stark, R., Grzelak, M. & Hadfield, J. RNA sequencing: the teenage years. *Nat. Rev. Genet.* **20**, 631–656 (2019). 31341269 10.1038/s41576-019-0150-2

[3] Glen, A. C. Measurement of DNA and RNA in human peripheral blood lymphocytes. *Clin. Chem.* **13**, 299–313 (1967). 6036715

[4] Rifai, N. *Tietz Textbook of Laboratory Medicine* (Elsevier, 2022).

[5] Byron, S. A., Van Keuren-Jensen, K. R., Engelthaler, D. M., Carpten, J. D. & Craig, D. W. Translating RNA sequencing into clinical diagnostics: opportunities and challenges. *Nat. Rev. Genet.* **17**, 257–271 (2016). 26996076 10.1038/nrg.2016.10 PMC7097555

[6] Fresard, L. et al. Identification of rare-disease genes using blood transcriptome sequencing and large control cohorts. *Nat. Med.* **25**, 911–919 (2019). 31160820 10.1038/s41591-019-0457-8 PMC6634302

[7] Cummings, B. B. et al. Improving genetic diagnosis in Mendelian disease with transcriptome sequencing. *Sci. Transl. Med.*10.1126/scitranslmed.aal5209 (2017). 10.1126/scitranslmed.aal5209 PMC5548421 28424332

[8] Best, M. G. et al. RNA-seq of tumor-educated platelets enables blood-based pan-cancer, multiclass, and molecular pathway cancer diagnostics. *Cancer Cell* **28**, 666–676 (2015). 26525104 10.1016/j.ccell.2015.09.018 PMC4644263

[9] Zuckerman, N. S. et al. Altered local and systemic immune profiles underlie lymph node metastasis in breast cancer patients. *Int. J. Cancer* **132**, 2537–2547 (2013). 23136075 10.1002/ijc.27933 PMC3609917

[10] Dumeaux, V. et al. Interactions between the tumor and the blood systemic response of breast cancer patients. *PLoS Comput. Biol.* **13**, e1005680 (2017). 28957325 10.1371/journal.pcbi.1005680 PMC5619688

[11] Bernardes, J. P. et al. Longitudinal multi-omics analyses identify responses of megakaryocytes, erythroid cells, and plasmablasts as hallmarks of severe COVID-19. *Immunity* **53**, 1296–1314.e9 (2020). 33296687 10.1016/j.immuni.2020.11.017 PMC7689306

[12] Hasler, R. et al. Uncoupling of mucosal gene regulation, mRNA splicing and adherent microbiota signatures in inflammatory bowel disease. *Gut* **66**, 2087–2097 (2017). 27694142 10.1136/gutjnl-2016-311651 PMC5749366

[13] Juzenas, S. et al. Detailed transcriptional landscape of peripheral blood points to increased neutrophil activation in treatment-naive inflammatory bowel disease. *J. Crohns Colitis* **16**, 1097–1109 (2022). 35022690 10.1093/ecco-jcc/jjac003 PMC9351981

[14] Warnat-Herresthal, S. et al. Swarm learning for decentralized and confidential clinical machine learning. *Nature* **594**, 265–270 (2021). 34040261 10.1038/s41586-021-03583-3 PMC8189907

[15] Denson, L. A. et al. Clinical and genomic correlates of neutrophil reactive oxygen species production in pediatric patients with Crohn’s disease. *Gastroenterology* **154**, 2097–2110 (2018). 29454792 10.1053/j.gastro.2018.02.016 PMC5985211

[16] Anderson, S. T. et al. Diagnosis of childhood tuberculosis and host RNA expression in Africa. *N. Engl. J. Med.* **370**, 1712–1723 (2014). 24785206 10.1056/NEJMoa1303657 PMC4069985

[17] Beck, L. A. et al. Dupilumab treatment in adults with moderate-to-severe atopic dermatitis. *N. Engl. J. Med.* **371**, 130–139 (2014). 25006719 10.1056/NEJMoa1314768

[18] Orange, D. E. et al. RNA identification of PRIME cells predicting rheumatoid arthritis flares. *N. Engl. J. Med.* **383**, 218–228 (2020). 32668112 10.1056/NEJMoa2004114 PMC7546156

[19] Davenport, E. E. et al. Genomic landscape of the individual host response and outcomes in sepsis: a prospective cohort study. *Lancet Respir. Med.* **4**, 259–271 (2016). 26917434 10.1016/S2213-2600(16)00046-1 PMC4820667

[20] Sanchez-Pino, M. D. et al. Increased inflammatory low-density neutrophils in severe obesity and effect of bariatric surgery: results from case-control and prospective cohort studies. *EBioMedicine* **77**, 103910 (2022). 35248994 10.1016/j.ebiom.2022.103910 PMC8897585

[21] Shigemizu, D. et al. Identification of potential blood biomarkers for early diagnosis of Alzheimer’s disease through RNA sequencing analysis. *Alzheimers Res. Ther.* **12**, 87 (2020). 32677993 10.1186/s13195-020-00654-x PMC7367375

[22] Craig, D. W. et al. RNA sequencing of whole blood reveals early alterations in immune cells and gene expression in Parkinson’s disease. *Nat. Aging* **1**, 734–747 (2021). 37117765 10.1038/s43587-021-00088-6

[23] Andrade-Navarro, M. A. et al. RNA sequencing of human peripheral blood cells indicates upregulation of immune-related genes in Huntington’s disease. *Front. Neurol.* **11**, 573560 (2020). 33329316 10.3389/fneur.2020.573560 PMC7731869

[24] Xiong, Y. et al. Transcriptomic characteristics of bronchoalveolar lavage fluid and peripheral blood mononuclear cells in COVID−19 patients. *Emerg. Microbes Infect.* **9**, 761–770 (2020). 32228226 10.1080/22221751.2020.1747363 PMC7170362

[25] Schulte-Schrepping, J. et al. Severe COVID−19 is marked by a dysregulated myeloid cell compartment. *Cell* **182**, 1419–1440 e1423 (2020). 32810438 10.1016/j.cell.2020.08.001 PMC7405822

[26] Wilk, A. J. et al. A single-cell atlas of the peripheral immune response in patients with severe COVID-19. *Nat. Med.* **26**, 1070–1076 (2020). 32514174 10.1038/s41591-020-0944-y PMC7382903

[27] Aschenbrenner, A. C. et al. Disease severity-specific neutrophil signatures in blood transcriptomes stratify COVID-19 patients. *Genome Med.* **13**, 7 (2021). 33441124 10.1186/s13073-020-00823-5 PMC7805430

[28] Rondina, M. T. et al. Longitudinal RNA-seq analysis of the repeatability of gene expression and splicing in human platelets identifies a platelet SELP splice QTL. *Circ. Res.* **126**, 501–516 (2020). 31852401 10.1161/CIRCRESAHA.119.315215 PMC7323475

[29] Schussler-Fiorenza Rose, S. M. et al. A longitudinal big data approach for precision health. *Nat. Med.* **25**, 792–804 (2019). 31068711 10.1038/s41591-019-0414-6 PMC6713274

[30] Sailani, M. R. et al. Deep longitudinal multiomics profiling reveals two biological seasonal patterns in California. *Nat. Commun.* **11**, 4933 (2020). 33004787 10.1038/s41467-020-18758-1 PMC7529769

[31] GTEx Consortium. Genetic effects on gene expression across human tissues. *Nature* **550**, 204–213 (2017). 29022597 10.1038/nature24277 PMC5776756

[32] Battle, A. et al. Characterizing the genetic basis of transcriptome diversity through RNA-sequencing of 922 individuals. *Genome Res.* **24**, 14–24 (2014). 24092820 10.1101/gr.155192.113 PMC3875855

[33] Lappalainen, T. et al. Transcriptome and genome sequencing uncovers functional variation in humans. *Nature* **501**, 506–511 (2013). 24037378 10.1038/nature12531 PMC3918453

[34] Pickrell, J. K. et al. Understanding mechanisms underlying human gene expression variation with RNA sequencing. *Nature* **464**, 768–772 (2010). 20220758 10.1038/nature08872 PMC3089435

[35] Whitney, A. R. et al. Individuality and variation in gene expression patterns in human blood. *Proc. Natl. Acad. Sci. USA* **100**, 1896–1901 (2003). 12578971 10.1073/pnas.252784499 PMC149930

[36] Wright, F. A. et al. Heritability and genomics of gene expression in peripheral blood. *Nat. Genet.* **46**, 430–437 (2014). 24728292 10.1038/ng.2951 PMC4012342

[37] Falony, G. et al. Population-level analysis of gut microbiome variation. *Science* **352**, 560–564 (2016). 27126039 10.1126/science.aad3503

[38] Liquet, B., Le Cao, K. A., Hocini, H. & Thiebaut, R. A novel approach for biomarker selection and the integration of repeated measures experiments from two assays. *BMC Bioinforma.* **13**, 325 (2012). 10.1186/1471-2105-13-325 PMC3627901 23216942

[39] Risso, D., Ngai, J., Speed, T. P. & Dudoit, S. Normalization of RNA-seq data using factor analysis of control genes or samples. *Nat. Biotechnol.* **32**, 896–902 (2014). 25150836 10.1038/nbt.2931 PMC4404308

[40] Chiang, E. Y. et al. CD96 functions as a co-stimulatory receptor to enhance CD8(+) T cell activation and effector responses. *Eur. J. Immunol.* **50**, 891–902 (2020). 32043568 10.1002/eji.201948405

[41] Carroll, D. J. et al. Sialic acid-binding immunoglobulin-like lectin 8 (Siglec-8) is an activating receptor mediating beta(2)-integrin-dependent function in human eosinophils. *J. Allergy Clin. Immunol.* **141**, 2196–2207 (2018). 28888781 10.1016/j.jaci.2017.08.013 PMC5839929

[42] Yanai, I. et al. Genome-wide midrange transcription profiles reveal expression level relationships in human tissue specification. *Bioinformatics* **21**, 650–659 (2005). 15388519 10.1093/bioinformatics/bti042

[43] Frankish, A. et al. GENCODE reference annotation for the human and mouse genomes. *Nucleic Acids Res.* **47**, D766–D773 (2019). 30357393 10.1093/nar/gky955 PMC6323946

[44] Newman, A. M. et al. Determining cell type abundance and expression from bulk tissues with digital cytometry. *Nat. Biotechnol.* **37**, 773–782 (2019). 31061481 10.1038/s41587-019-0114-2 PMC6610714

[45] Lloyd-Jones, L. R. et al. The genetic architecture of gene expression in peripheral blood. *Am. J. Hum. Genet.* **100**, 228–237 (2017). 28065468 10.1016/j.ajhg.2016.12.008 PMC5294670

[46] Gonzalez-Porta, M., Calvo, M., Sammeth, M. & Guigo, R. Estimation of alternative splicing variability in human populations. *Genome Res.* **22**, 528–538 (2012). 22113879 10.1101/gr.121947.111 PMC3290788

[47] Baruah, S. et al. Identification of a novel splice variant isoform of TREM-1 in human neutrophil granules. *J. Immunol.* **195**, 5725–5731 (2015). 26561551 10.4049/jimmunol.1402713 PMC4670805

[48] Langfelder, P. & Horvath, S. WGCNA: an R package for weighted correlation network analysis. *BMC Bioinforma.* **9**, 559 (2008). 10.1186/1471-2105-9-559 PMC2631488 19114008

[49] Mishra, N. et al. Longitudinal multi-omics analysis identifies early blood-based predictors of anti-TNF therapy response in inflammatory bowel disease. *Genome Med.* **14**, 110 (2022). 36153599 10.1186/s13073-022-01112-z PMC9509553

[50] Prebensen, C. et al. Longitudinal whole blood transcriptomic analysis characterizes neutrophil activation and interferon signaling in moderate and severe COVID-19. *Sci. Rep.* **13**, 10368 (2023). 37365222 10.1038/s41598-023-37606-y PMC10293211

[51] Baumgart, M. et al. Longitudinal RNA-seq analysis of vertebrate aging identifies mitochondrial complex I as a Small-molecule-sensitive Modifier Of Lifespan. *Cell Syst.* **2**, 122–132 (2016). 27135165 10.1016/j.cels.2016.01.014

[52] Medina, M. A. et al. Longitudinal transcriptional changes reveal genes from the natural killer cell-mediated cytotoxicity pathway as critical players underlying COVID-19 progression. *Elife*10.7554/eLife.94242 (2024). 10.7554/eLife.94242 PMC11521369 39470726

[53] Pavlovic, N., Krizanac, M., Kumric, M., Vukojevic, K. & Bozic, J. Mitochondrial dysfunction: the silent catalyst of kidney disease progression. *Cells*10.3390/cells14110794 (2025). 10.3390/cells14110794 PMC12153900 40497970

[54] Lovisa, S., Zeisberg, M. & Kalluri, R. Partial epithelial-to-mesenchymal transition and other new mechanisms of kidney fibrosis. *Trends Endocrinol. Metab.* **27**, 681–695 (2016). 27372267 10.1016/j.tem.2016.06.004

[55] Flaquer, A. et al. Mitochondrial GWA analysis of lipid profile identifies genetic variants to be associated with HDL cholesterol and triglyceride levels. *PLoS ONE* **10**, e0126294 (2015). 25945934 10.1371/journal.pone.0126294 PMC4422732

[56] The GTEx Consortium. The GTEx consortium atlas of genetic regulatory effects across human tissues. *Science* **369**, 1318–1330 (2020). 32913098 10.1126/science.aaz1776 PMC7737656

[57] Vosa, U. et al. Large-scale cis- and trans-eQTL analyses identify thousands of genetic loci and polygenic scores that regulate blood gene expression. *Nat. Genet* **53**, 1300–1310 (2021). 34475573 10.1038/s41588-021-00913-z PMC8432599

[58] Chen, L. et al. Alterations in gut microbiota and host transcriptome of patients with coronary artery disease. *BMC Microbiol.* **23**, 320 (2023). 37924005 10.1186/s12866-023-03071-w PMC10623719

[59] Ostrowski, J. et al. Redefining the practical utility of blood transcriptome biomarkers in inflammatory bowel diseases. *J. Crohns Colitis* **13**, 626–633 (2019). 30541017 10.1093/ecco-jcc/jjy205 PMC6486489

[60] Mo, A. et al. Disease-specific regulation of gene expression in a comparative analysis of juvenile idiopathic arthritis and inflammatory bowel disease. *Genome Med.* **10**, 48 (2018). 29950172 10.1186/s13073-018-0558-x PMC6020373

[61] Beretta, L. et al. Genome-wide whole blood transcriptome profiling in a large European cohort of systemic sclerosis patients. *Ann. Rheum. Dis.* **79**, 1218–1226 (2020). 32561607 10.1136/annrheumdis-2020-217116 PMC7456554

[62] Li, H., Wang, X., Yang, Q., Cheng, L. & Zeng, H. L. Identification of iron metabolism-related genes as diagnostic signatures in sepsis by blood transcriptomic analysis. *Open Life Sci.* **18**, 20220549 (2023). 36820206 10.1515/biol-2022-0549 PMC9938542

[63] Crow, M., Lim, N., Ballouz, S., Pavlidis, P. & Gillis, J. Predictability of human differential gene expression. *Proc. Natl. Acad. Sci. USA* **116**, 6491–6500 (2019). 30846554 10.1073/pnas.1802973116 PMC6442595

[64] Aaroe, J. et al. Gene expression profiling of peripheral blood cells for early detection of breast cancer. *Breast Cancer Res.* **12**, R7 (2010). 20078854 10.1186/bcr2472 PMC2880427

[65] Alonso, V. et al. Gene expression profile in the peripheral blood of patients with prostate cancer and benign prostatic hyperplasia. *Cancer Detect Prev.* **32**, 336–337 (2009). 19026495 10.1016/j.cdp.2008.10.001

[66] Netea, M. G. et al. Understanding human immune function using the resources from the Human Functional Genomics Project. *Nat. Med* **22**, 831–833 (2016). 27490433 10.1038/nm.4140

[67] Jaitin, D. A. et al. Massively parallel single-cell RNA-seq for marker-free decomposition of tissues into cell types. *Science* **343**, 776–779 (2014). 24531970 10.1126/science.1247651 PMC4412462

[68] Mass, E. et al. Specification of tissue-resident macrophages during organogenesis. *Science*10.1126/science.aaf4238 (2016). 10.1126/science.aaf4238 PMC5066309 27492475

[69] Paul, F. et al. Transcriptional heterogeneity and lineage commitment in myeloid progenitors. *Cell* **163**, 1663–1677 (2015). 26627738 10.1016/j.cell.2015.11.013

[70] Stegle, O., Parts, L., Piipari, M., Winn, J. & Durbin, R. Using probabilistic estimation of expression residuals (PEER) to obtain increased power and interpretability of gene expression analyses. *Nat. Protoc.* **7**, 500–507 (2012). 22343431 10.1038/nprot.2011.457 PMC3398141

[71] Rinn, J. L. & Snyder, M. Sexual dimorphism in mammalian gene expression. *Trends Genet.* **21**, 298–305 (2005). 15851067 10.1016/j.tig.2005.03.005

[72] Yang, X. et al. Tissue-specific expression and regulation of sexually dimorphic genes in mice. *Genome Res.* **16**, 995–1004 (2006). 16825664 10.1101/gr.5217506 PMC1524872

[73] Jansen, R. et al. Sex differences in the human peripheral blood transcriptome. *BMC Genomics* **15**, 33 (2014). 24438232 10.1186/1471-2164-15-33 PMC3904696

[74] Ma, H. et al. Altered cytokine gene expression in peripheral blood monocytes across the menstrual cycle in primary dysmenorrhea: a case-control study. *PLoS ONE* **8**, e55200 (2013). 23390521 10.1371/journal.pone.0055200 PMC3563666

[75] Dopico, X. C. et al. Widespread seasonal gene expression reveals annual differences in human immunity and physiology. *Nat. Commun.* **6**, 7000 (2015). 25965853 10.1038/ncomms8000 PMC4432600

[76] Ferraro, N. M. et al. Transcriptomic signatures across human tissues identify functional rare genetic variation. *Science*10.1126/science.aaz5900 (2020). 10.1126/science.aaz5900 PMC7646251 32913073

[77] Mule, M. P. et al. Integrating population and single-cell variations in vaccine responses identifies a naturally adjuvanted human immune setpoint. *Immunity* **57**, 1160–1176 e1167 (2024). 38697118 10.1016/j.immuni.2024.04.009

[78] Sparks, R. et al. A unified metric of human immune health. *Nat. Med.* **30**, 2461–2472 (2024). 38961223 10.1038/s41591-024-03092-6 PMC12183718

[79] Cheng, J. et al. Transcriptional maps of 10 human chromosomes at 5-nucleotide resolution. *Science* **308**, 1149–1154 (2005). 15790807 10.1126/science.1108625

[80] Yang, L., Duff, M. O., Graveley, B. R., Carmichael, G. G. & Chen, L. L. Genomewide characterization of non-polyadenylated RNAs. *Genome Biol.* **12**, R16 (2011). 21324177 10.1186/gb-2011-12-2-r16 PMC3188798

[81] Ewels, P. A. et al. The nf-core framework for community-curated bioinformatics pipelines. *Nat. Biotechnol.* **38**, 276–278 (2020). 32055031 10.1038/s41587-020-0439-x

[82] Dobin, A. et al. STAR: ultrafast universal RNA-seq aligner. *Bioinformatics* **29**, 15–21 (2013). 23104886 10.1093/bioinformatics/bts635 PMC3530905

[83] Liao, Y., Smyth, G. K. & Shi, W. featureCounts: an efficient general purpose program for assigning sequence reads to genomic features. *Bioinformatics* **30**, 923–930 (2014). 24227677 10.1093/bioinformatics/btt656

[84] Hoffman, G. E. & Schadt, E. E. variancePartition: interpreting drivers of variation in complex gene expression studies. *BMC Bioinforma.* **17**, 483 (2016). 10.1186/s12859-016-1323-z PMC5123296 27884101

[85] Pertea, M. et al. StringTie enables improved reconstruction of a transcriptome from RNA-seq reads. *Nat. Biotechnol.* **33**, 290–295 (2015). 25690850 10.1038/nbt.3122 PMC4643835

[86] Soneson, C., Love, M. I. & Robinson, M. D. Differential analyses for RNA-seq: transcript-level estimates improve gene-level inferences. *F1000Res.* **4**, 1521 (2015). 26925227 10.12688/f1000research.7563.1 PMC4712774

[87] Nowicka, M. & Robinson, M. D. DRIMSeq: a Dirichlet-multinomial framework for multivariate count outcomes in genomics. *F1000Res.* **5**, 1356 (2016). 28105305 10.12688/f1000research.8900.1 PMC5200948

[88] Delaneau, O. et al. A complete tool set for molecular QTL discovery and analysis. *Nat. Commun.* **8**, 15452 (2017). 28516912 10.1038/ncomms15452 PMC5454369

[89] Smid, M. et al. Gene length corrected trimmed mean of M-values (GeTMM) processing of RNA-seq data performs similarly in intersample analyses while improving intrasample comparisons. *BMC Bioinforma.* **19**, 236 (2018). 10.1186/s12859-018-2246-7 PMC6013957 29929481

[90] Aulchenko, Y. S., Ripke, S., Isaacs, A. & van Duijn, C. M. GenABEL: an R library for genome-wide association analysis. *Bioinformatics* **23**, 1294–1296 (2007). 17384015 10.1093/bioinformatics/btm108

[91] Wang, L., Wang, S. & Li, W. RSeQC: quality control of RNA-seq experiments. *Bioinformatics* **28**, 2184–2185 (2012). 22743226 10.1093/bioinformatics/bts356

[92] Mallick, H. et al. Multivariable association discovery in population-scale meta-omics studies. *PLoS Comput. Biol.* **17**, e1009442 (2021). 34784344 10.1371/journal.pcbi.1009442 PMC8714082

[93] Pinheiro, J. C. & Bates, D. M. Mixed-effects models in S and S-PLUS 10.1007/b98882 (2000).

[94] Mishra, N., Kimmig, F. & Rosenstiel, P. Large-scale analysis of temporal gene expression variation in peripheral blood (code) 10.5281/zenodo.19697118 (2026). 10.1038/s41467-026-73218-6 PMC13392353 42215465

