# Supplementary material for: Large-scale analysis of temporal gene expression variation in peripheral blood
Source: Nat Commun. 2026 May 29;17:6992. doi: 10.1038/s41467-026-73218-6 (PMC13392353; doi:10.1038/s41467-026-73218-6)
Supplement: Supplementary file 1 — Supplementary Information [file 41467_2026_73218_MOESM1_ESM.pdf]

Supplementary Information

Large-scale analysis of temporal gene expression variation in peripheral blood

Mishra et al.

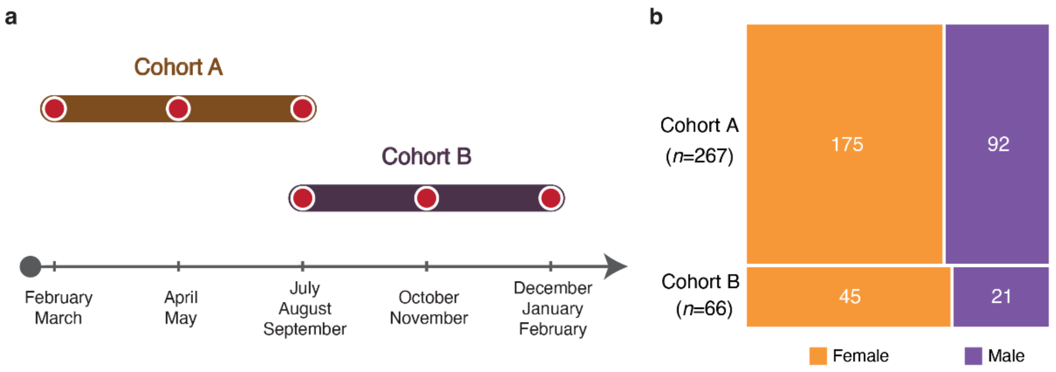

c

	Metadata examples	Distributions Individuals ($N = 333$)
Anthropometrics	Age (years) Median [range]	48 [19 - 79], $N = 333$
	Biological Sex Number, percent	Females (220, 66.1%), Males (113, 33.9%)
	BMI Median [range]	Females (23.88 [17.61 - 49.10], $N = 211$), Males (25.21 [19.70 - 36.93], $N = 113$)
Sampling	Blood samples Number, percent	3 time points (333, 100%)
	RNA-sequencing Number, percent	3 time points (305, 91.6%), 2 time points (27, 8.1%), 1 time point (1, 0.3%)
Health records and surveys	Self-reported medical conditions Number, percent	Chronic inflammatory diseases: Yes (41, 12.3%)<br>Cancer (non-active): Yes (13, 3.9%)<br>Depression: Yes (38, 11.4%)<br>Pulmonary disease (asthma): Yes (19, 5.7%)<br>Diabetes: Yes (5, 1.5%)<br>Other (eg, acne, gastroesophageal reflux, arthrosis): Yes (190, 57.1%)
	Hay fever Number, percent	Yes (59, 17.7%)

	Metadata examples	Distributions Samples ($N = 970$)
Clinical laboratory profiling	hsCRP (mg L^{-1}) Median [range]	0.90 [0.30 - 63.40], $N = 962$
	Creatinine (mg dL^{-1}) Median [range]	0.81 [0.41 - 1.61], $N = 961$
	Hemoglobin (g dL^{-1}) Median [range]	Females (13.50 [7.50 - 15.80], $N = 635$), Males (15.00 [13.00 - 18.20], $N = 329$)
Sampling	Time of sampling Median [range]	9AM [6:00am - 21:09pm], $N = 777$
	Season of sampling Number, percent	Winter (321, 33.1%), Spring (265, 27.3%), Summer (317, 32.7%), Autumn (67, 6.9%)
Health records and surveys	Acute infection during sampling Number, percent	Yes (44, 4.9%), No (855, 95.1%), $N = 899$

Supplementary Fig. 1: Study design and cohort characteristics. a, Schematic overview of the study design. **b,** Mosaic plot showing the proportion of females and males in the two cohorts. **c,** Categories of collected demographic and clinical features. Selected examples and their distribution in the cohort are listed for each category. Source data are provided as a Source Data file.

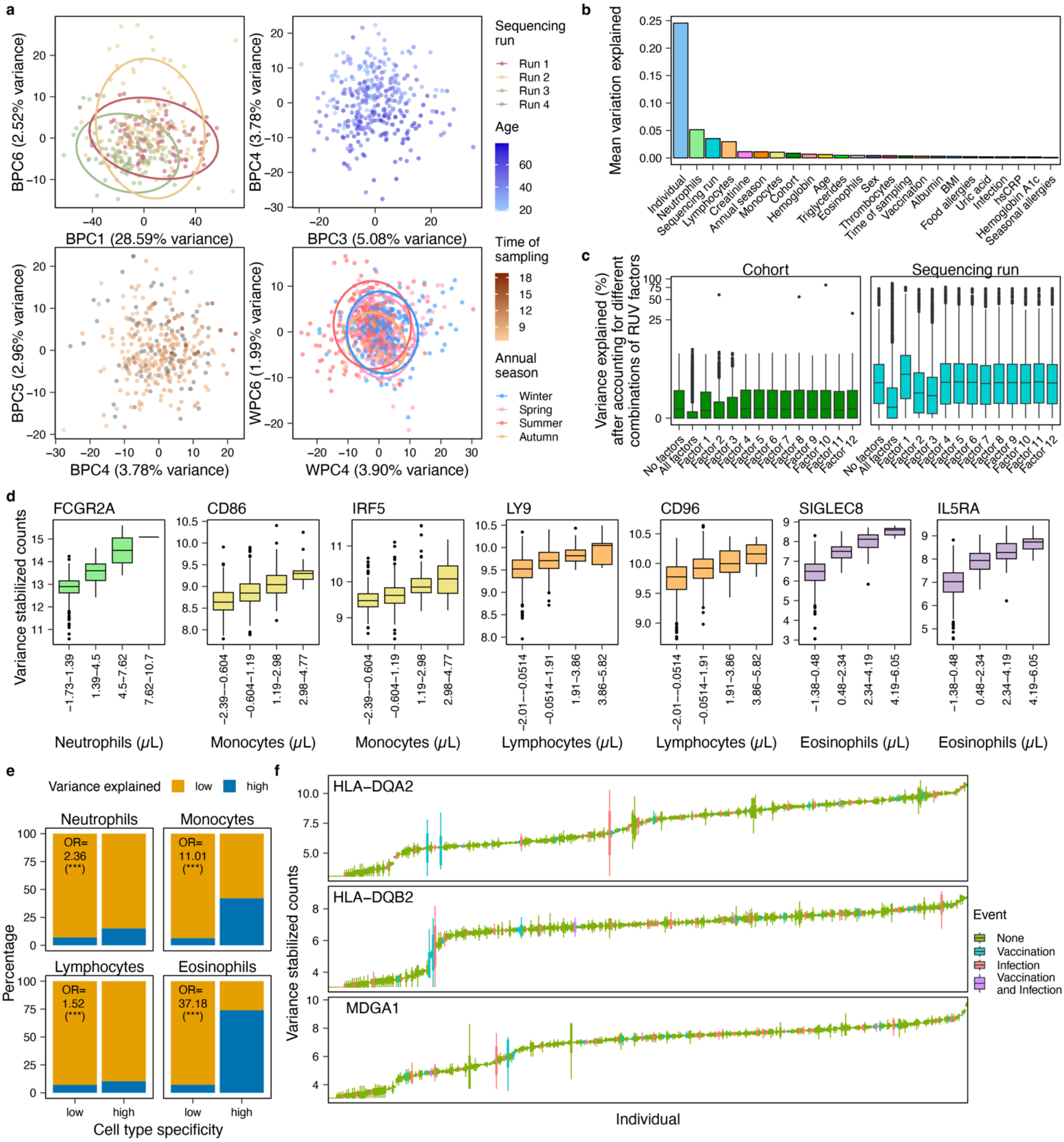

Supplementary Fig. 2: Technical and biological sources of expression variance. a, Between- and within-individual PCA plots for all samples based on genes that passed the filtering cutoff (n = 14,166, see Methods, coloured by sequencing run (top left), age (top right), diurnal time of sampling (bottom left), and annual season (bottom right). **b,** Mean proportion of variance in gene expression explained by different clinical and technical parameters across all genes (n = 13,702). **c,** Percentage of variance in gene expression (n = 13,702) explained by sequencing run (left) and cohort (right) after accounting for no, all, or individual RUV factors. Each data point represents a gene. Box plots show median (centre line), interquartile range (IQR; box), and whiskers extend to 1.5xIQR. Points represent outliers beyond the whiskers. **d,** Expression levels of selected genes with variance explained by different blood cell counts across

samples stratified by cell type abundance. Boxplots are defined as in (c). **e,** Odds ratios estimated using Fisher's exact test (two-sided) for enrichment of cell type markers in top 1000 genes ranked by the variance explained by different blood cell counts. *FDR<0.05, **FDR<0.01, ***FDR<0.0001. **f,** Expression levels of selected genes with variance explained by individual identity across multiple samples per individual. Individuals are coloured by reported infection or vaccination events. Each data point represents a biological replicate. Box plots show median (centre line), interquartile range (IQR; box), and whiskers extend to 1.5xIQR. Source data are provided as a Source Data file.

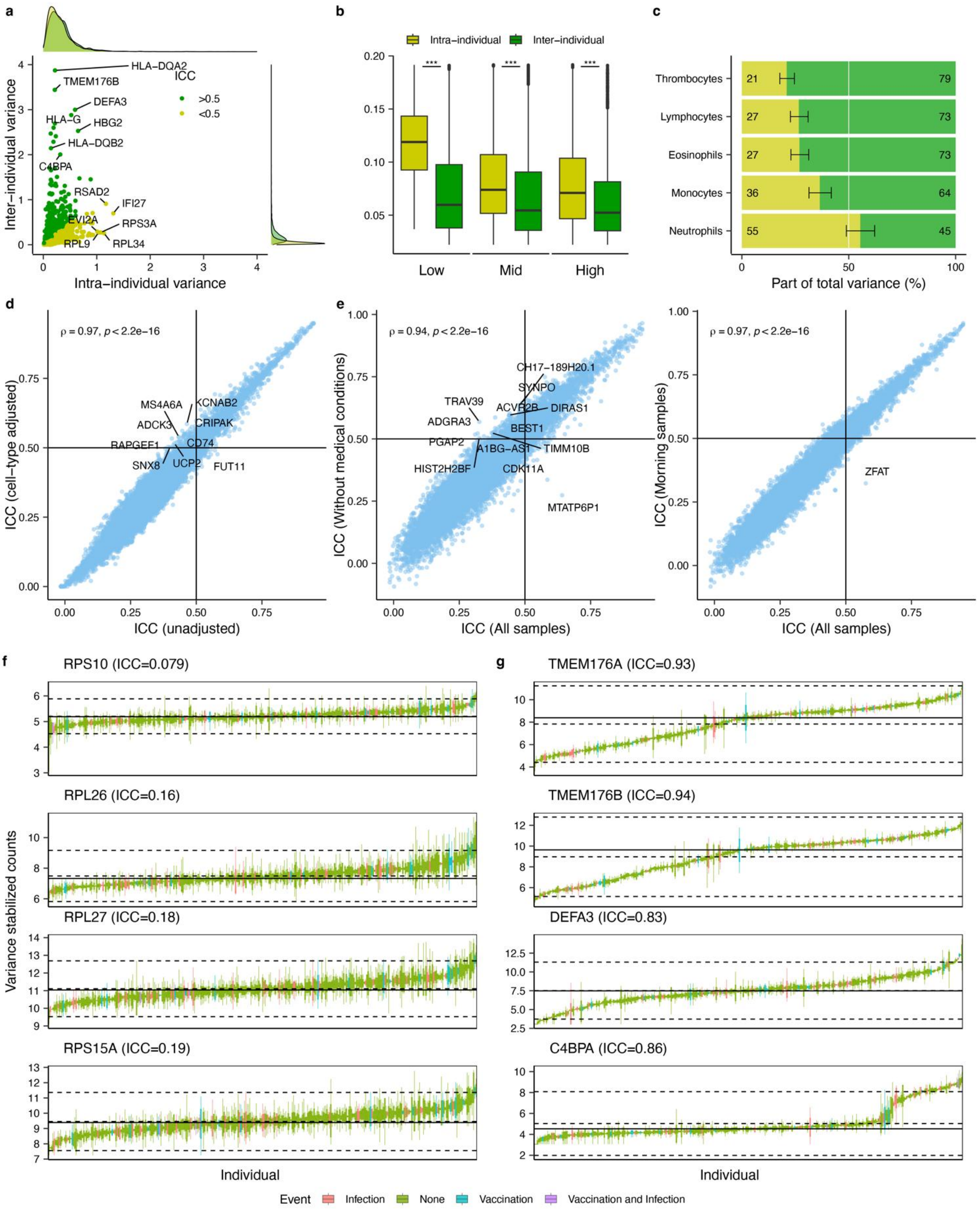

Supplementary Fig. 3: Gene-level intra- and inter-individual variance. a, Scatterplot with kernel density estimates of inter- and intra-individual variance for protein-coding genes (n = 11,641). Genes with ICC > 0.5 are denoted in green and those with ICC < 0.5 are denoted in yellow. **b,** Intra- and inter-individual variance for protein-coding genes stratified by low (n = 3,881), medium (n = 3,880), and high (n = 3,880) expression levels. Each data point represents a gene. Boxplots show median (centre line), interquartile range (IQR; box), and whiskers extend to 1.5xIQR. Points

represent outliers beyond the whiskers. Paired Mann-Whitney *U* test (two-sided) was used, and *p*-values were adjusted for multiple tests using Benjamini-Hochberg false discovery rate (FDR); *FDR<0.05, **FDR<0.01, ***FDR<0.0001. **c**, Proportion of intra- and inter-individual variance contributing to total variance in different blood cell counts. Error bars correspond to 95% confidence intervals of the ICC. **d,** Comparison of ICC values estimated with unadjusted and cell-type adjusted models. Each data point represents a gene. Correlation coefficients are calculated using Spearman's method. **e**, Comparison of ICC values between all samples and healthy samples (left) and between all samples and samples collected in the morning (right) (*n* = 13,702). Each data point represents a gene. Correlation coefficients are calculated using Spearman's method. **f-g**, Expression profiles of representative genes with low (**f**) and high (**g**) ICC values across multiple samples per individual. Individuals are coloured by reported infection or vaccination events. Each data point represents a biological replicate. Box plots show median (centre line), interquartile range (IQR; box), and whiskers extend to 1.5xIQR. Source data are provided as a Source Data file.

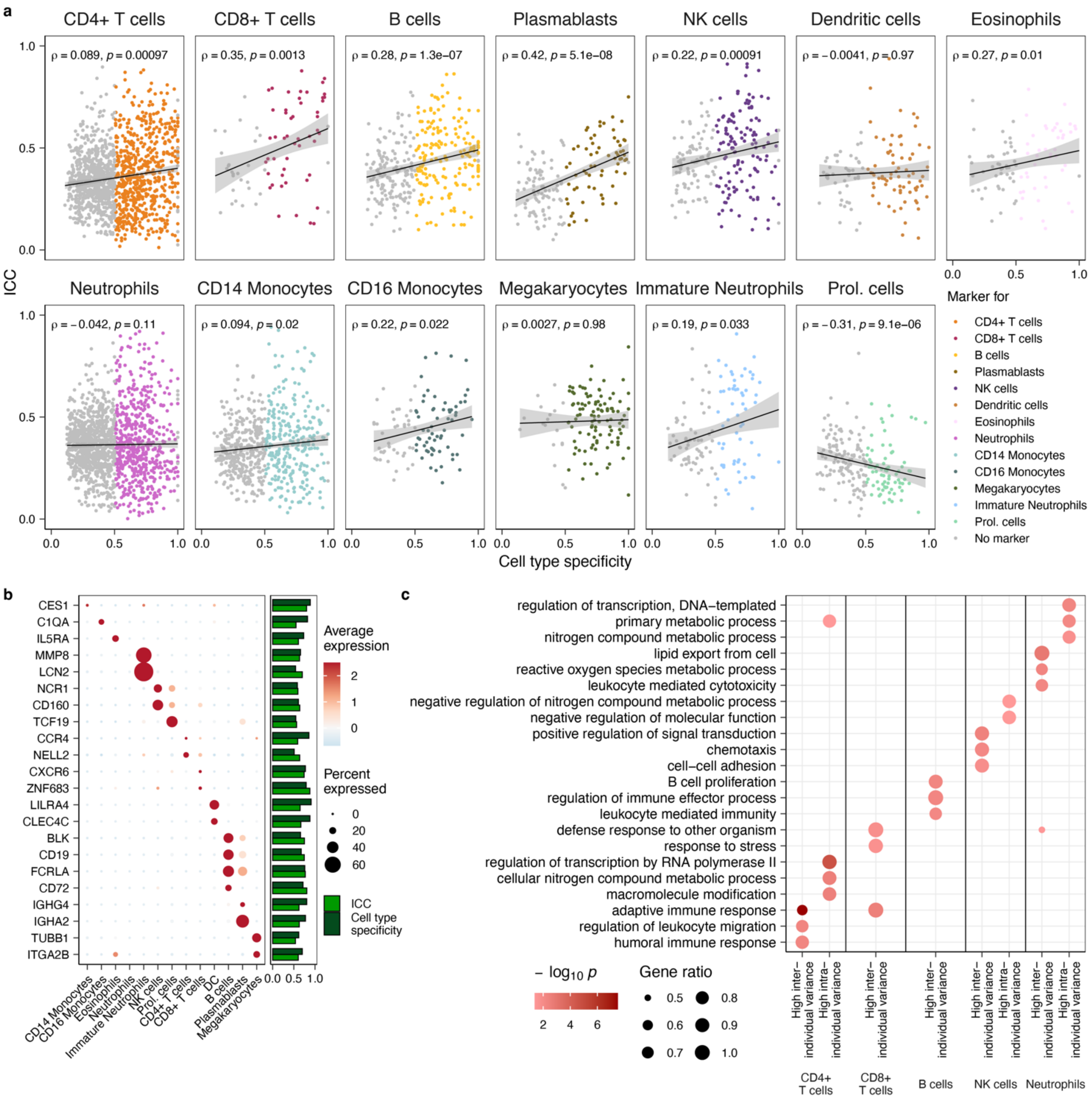

Supplementary Fig. 4: Cell-type specificity and expression stability. a, Scatter plots showing relationship between cell type specificity scores and ICC values of cell type specific genes across different cell types. Each data point represents a gene. Correlation coefficients are calculated using Spearman's method. Genes identified as cell type markers (see methods) are coloured. **b**, Dot plot of selected cell type marker genes with high ICC. Colour shows the scaled average expression, and point size represents the number of cells per group expressing the corresponding gene. Bar plots depict the ICC values and cell type specificity scores for each gene. **c**, GO terms enriched among gene sets classified based on ICC and cell type specificity. Dot size is proportional to the gene ratio and colour denotes enrichment *p*-value estimated using Fisher exact test (two-sided). Source data are provided as a Source Data file.

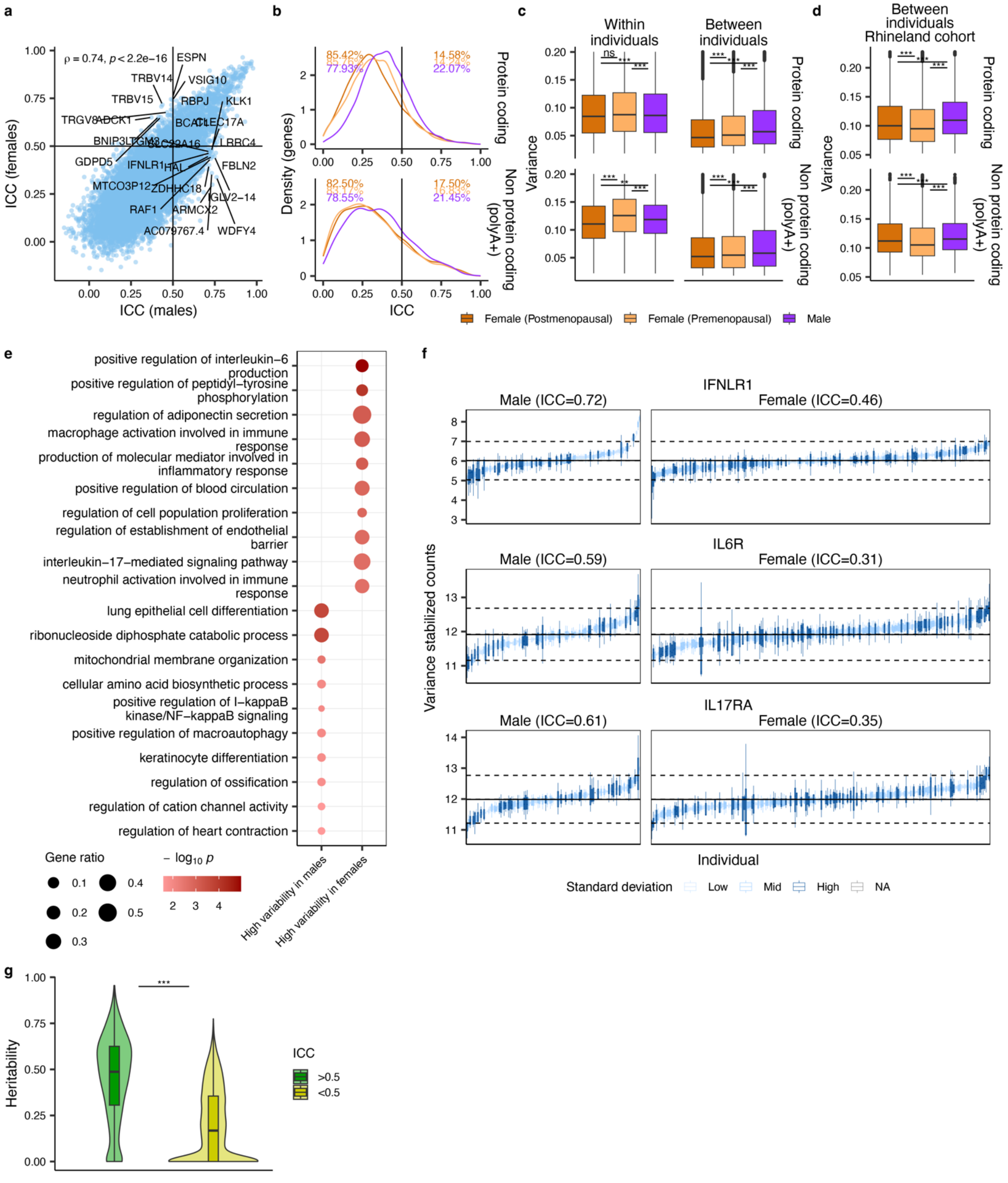

Supplementary Fig 5: Sex-associated differences in expression variability. a, Scatter plot showing comparison of ICC values between males and females (n = 14,146). Each data point represents a gene. Correlation coefficients are calculated using Spearman's method. **b**, Distribution of ICC values of protein-coding (n = 12,043) and non-protein-coding (polyA$^+$) (n = 2,103) genes in pre- (lightorange) and postmenopausal (darkorange) females and males (purple). **c**, Comparison of intra- and inter-individual variance in gene expression of protein-coding and non-protein-coding (polyA$^+$) genes across sex and menopausal status. Each data point represents a gene. Boxplots show median (centre line), interquartile range (IQR; box), and whiskers

extend to 1.5xIQR. Points represent outliers beyond the whiskers. Paired Mann-Whitney *U* test (two-sided) was used, and *p*-values were adjusted for multiple tests using Benjamini-Hochberg false discovery rate (FDR); *FDR<0.05, **FDR<0.01, ***FDR<0.0001. **d**, Comparison of inter-individual variance of protein-coding (n = 10,264) and non-protein-coding (polyA$^+$) (*n* = 888) genes across sex and menopausal status in the validation cohort. Boxplots and statistical tests are defined as in (c). **e**, GO terms enriched among genes showing large sex-specific differences in ICC. Dot size is proportional to the gene ratio and colour corresponds to the *p*-value of enrichment estimated using Fisher exact test (two-sided). **f**, Expression profiles of example genes with high ICC in males but low ICC in females. Individuals are coloured by the standard deviation across different samples. Each data point represents a biological replicate. Box plots show median (centre line), interquartile range (IQR; box), and whiskers extend to 1.5xIQR. **g**, Heritability scores of genes with high inter- or intra-individual variance. Each data point represents a gene. Violin plots show the distribution of the data, with the width representing kernel density. The centre line indicates the median, the box denotes the interquartile range (IQR), whiskers extend to 1.5× the IQR. Mann-Whitney *U* test (two-sided); **p*<0.05, ***p*<0.01, ****p*<0.0001. Source data are provided as a Source Data file.

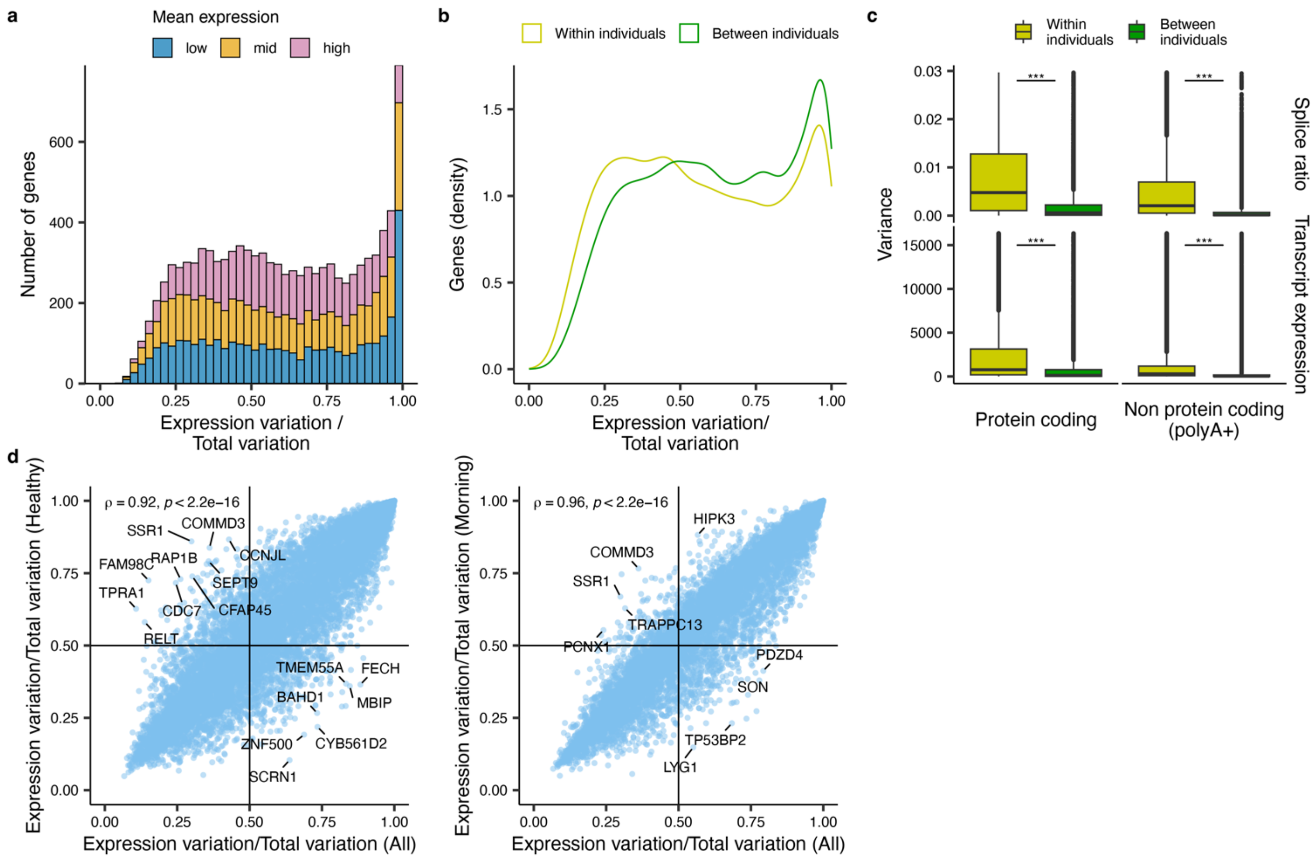

Supplementary Fig. 6: Splicing versus expression-level contributions to transcriptomic variability. a, Histograms showing the contribution of expression level variation to total variability stratified by mean expression level (*n* = 10,766). **b,** Density plots showing contribution of expression level variation within individuals to the total transcriptional variation within individuals (yellow) and of expression level variation between individuals to the total transcriptional variation between individuals (green) (*n* = 10,766). **c,** Intra- and inter-individual variance in transcript expression and splice ratios of protein-coding (*n* = 28,252) and non-protein- coding (polyA$^+$) (*n* = 31,189) transcripts. Each data point represents a transcript. Boxplots show median (centre line), interquartile range (IQR; box), and whiskers extend to 1.5xIQR. Points represent outliers beyond the whiskers. Paired Mann-Whitney *U* test (two-sided) was used, and *p*-values were adjusted for multiple tests using Benjamini-Hochberg false discovery rate (FDR); *FDR<0.05, **FDR<0.01, ***FDR<0.0001. **d,** Scatter plots comparing the contribution of expression level variation to the total variability between all samples and each of the sub-cohorts (*n* = 10,766). Each data point represents a gene. Correlation coefficients are calculated using Spearman's method. Source data are provided as a Source Data file.

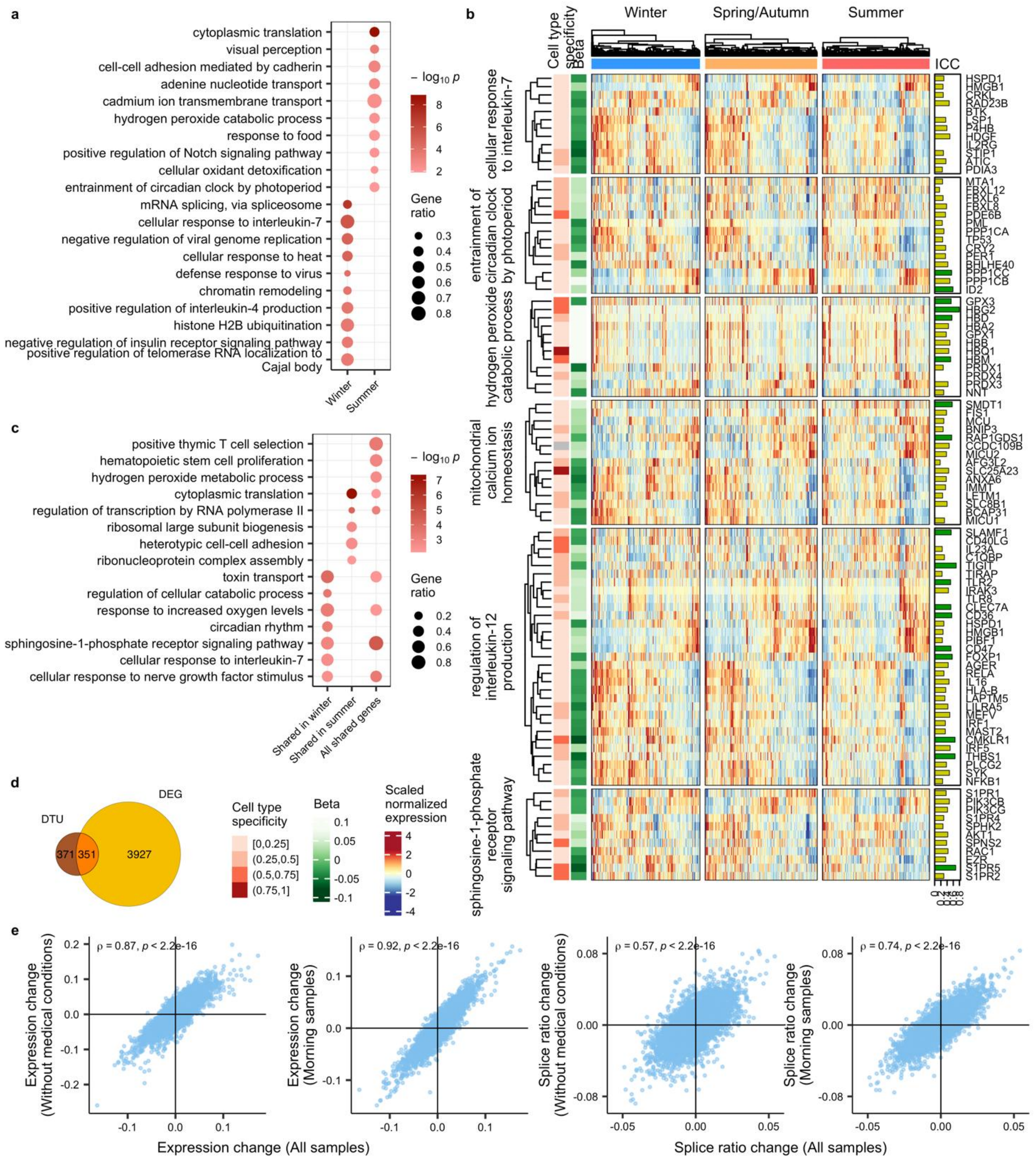

Supplementary Fig. 7: Seasonal variation analysis. a, GO terms enriched among genes with higher expression in winter and summer. Dot size is proportional to the gene ratio and color corresponds to the *p*-value of enrichment estimated using Fisher exact test (two-sided). **b,** Heatmap of seasonally differentially expressed genes enriched for selected biological processes. Row-wise z-scores of the normalized counts are plotted. **c**, GO terms enriched among seasonally regulated genes shared between the longitudinal and validation cohort. Dot size is proportional to the gene ratio and color denotes the enrichment *p*-value estimated using Fisher exact test (two-sided). **d**, Venn Diagram showing the overlap between genes with differential expression (DE) and differential transcript usage (DTU) across seasons. **e**, Scatter plots comparing seasonal change in gene expression and splice ratios between all samples and each of the sub-cohorts (n = 12,057 genes, n = 47,626 transcripts). Correlation coefficients are calculated using Spearman's method. Source data are provided as a Source Data file.

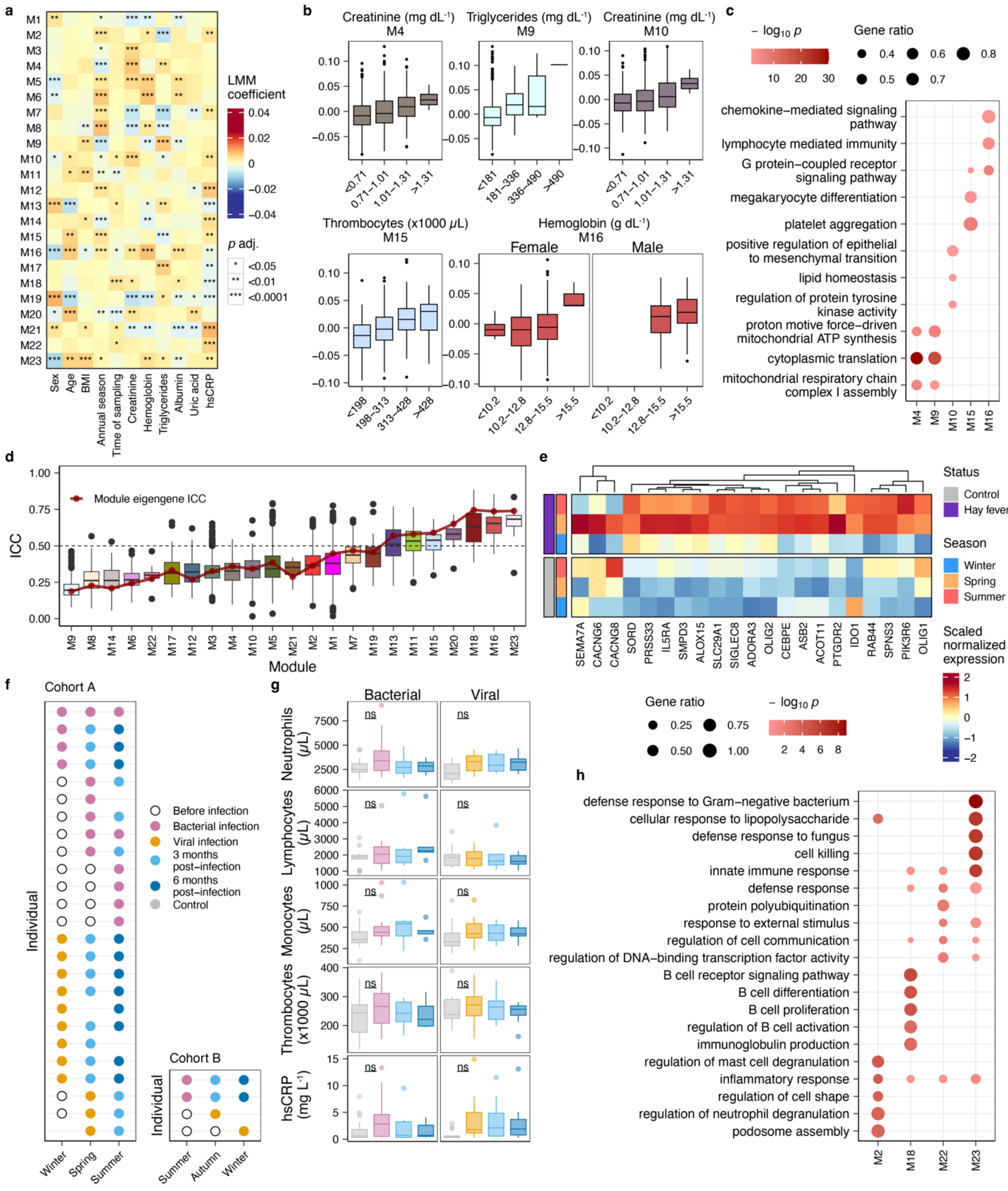

Supplementary Fig. 8: Co-expression modules and clinical associations. a, Heatmap of associations between module eigengenes (columns) and clinical parameters (rows) estimated using linear mixed-effects models (two-sided) adjusted for donor. *P*-values are corrected for multiple tests using Benjamini-Hochberg false discovery rate (FDR); *FDR<0.05, ** FDR <0.01, *** FDR <0.0001. **b,** Eigengene values of selected modules associated with clinical parameters. Each data point represents a biological sample. Boxplots show median (centre line), interquartile range (IQR; box), and whiskers extend to 1.5xIQR. Points represent outliers beyond the whiskers. **c,** GO terms enriched in selected modules associated with clinical parameters (M4, M9, M10, M15, and M16). Dot size is proportional to the gene ratio and colour corresponds to the enrichment *p*-value estimated using Fisher exact test

(two-sided). **d,** Distribution of ICC values for genes within each module. ICC values for module eigengenes are depicted by the maroon dots and line. Each data point represents a gene. Boxplots are defined as in (b). **e,** Heatmap of genes in module M20. Average row-wise z-scores of the normalized counts are plotted. **f,** Distribution of samples from individuals with a viral or bacterial infection at one of the timepoints separated by cohort. **g,** Blood cell counts and hsCRP levels in samples from individuals with infection and matched controls. Each data point represents a biological sample. Boxplots are defined as in (b). Mann-Whitney *U* test (two-sided) was used, and *p*-values were adjusted for multiple tests using Benjamini-Hochberg false discovery rate (FDR); ns FDR>0.5, *FDR<0.05, **FDR<0.01, ***FDR<0.0001. **h,** GO terms enriched among genes in infection-associated modules (M2, M18, M22 and M23). Dot size is proportional to the gene ratio and colour corresponds to the enrichment *p*-value estimated using Fisher exact test (two-sided). Source data are provided as a Source Data file.

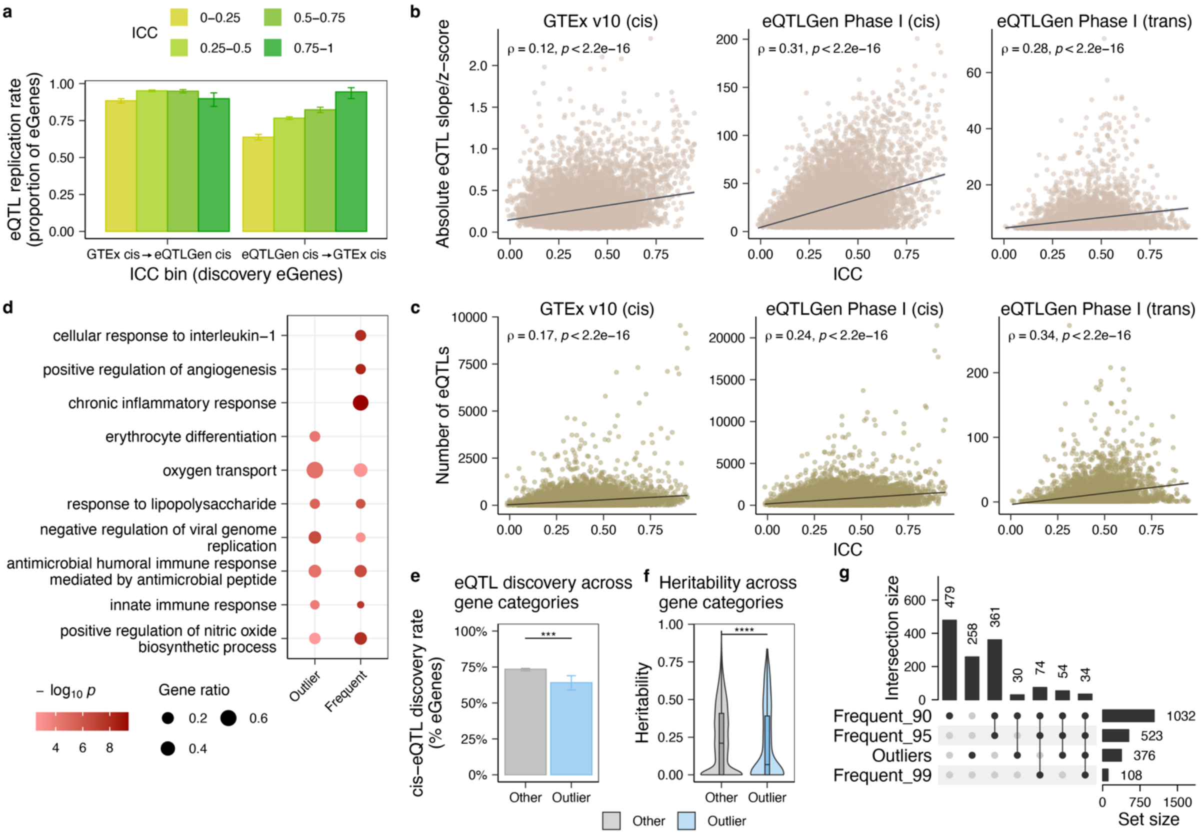

Supplementary Fig. 9: Impact of intra-individual variability on eQTL discovery and frequent differential expression. a, eQTL replication rates across ICC bins between GTEx v10 and eQTLGen Phase I cis-eQTL datasets. Bars represent the proportion of shared eGenes between two datasets within a given ICC bin. Error bars indicate 95% confidence intervals derived from a binomial test (two-sided). Each observation represents an aggregated proportion rather than individual gene. The number of genes contributing to each estimate ordered by increasing ICC bin are as follows: GTEx cis → eQTLGen cis (*n* = 1,918; 6,244; 1,534; 187), eQTLGen → cis GTEx cis (*n* = 2,658; 7,749; 1,769; 178). **b,** Scatter plots comparing ICC with eQTL effect sizes (absolute slope in GTEx, absolute z-score in eQTLGen). Each data point represents a gene. Correlation coefficients are calculated using Spearman's method. **c,** Scatter plots comparing ICC with number of eQTLs detected across datasets. Each data point represents a gene. Correlation coefficients are calculated using Spearman's method. **d,** GO terms enriched in "outlier genes" and "frequent DEGs" (DE prior rank cutoff 0.99). Dot size is proportional to the gene ratio and colour corresponds to the enrichment *p*-value estimated using Fisher exact test (two-sided). **e-f,** Comparison of cis-eQTL discovery rates (GTEX v10) and heritability between "outlier genes" and all other genes used in the analysis. Bars represent the proportion of eGenes in each category. Error bars indicate 95% confidence intervals derived from a binomial test (two-sided). Chi-square test (two-sided); ns *p*>0.5, **p*<0.05, ***p*<0.01, ****p*<0.0001 (**e**); Each data point represents a gene. Violin plots show the distribution of the data, with the width representing kernel density. The centre line indicates the median, the box denotes the interquartile range (IQR), whiskers extend to 1.5× the IQR. Mann-Whitney *U* test (two-sided);; ns *p*>0.5, **p*<0.05, ***p*<0.01, ****p*<0.0001 (**f**). **g,** UpSet plot showing intersections between outlier genes and "frequent DEGs" defined using DE prior rank cutoffs of 0.99 (Frequent_99), 0.95 (Frequent_95), and 0.90 (Frequent_90). Source data are provided as a Source Data file.
