# Supplementary material for: Large-scale analysis of temporal gene expression variation in peripheral blood
Source: Nat Commun. 2026 May 29;17:6992. doi: 10.1038/s41467-026-73218-6 (PMC13392353; doi:10.1038/s41467-026-73218-6)
Supplement: Supplementary file 2 — Description of Additional Supplementary Files [file 41467_2026_73218_MOESM2_ESM.pdf]

Description of Additional Supplementary Files

File Name: Supplementary Data 1

Description: Clinical and laboratory characteristics of samples and individuals used in different analyses of this study. Sheet 1: Description of tables. **Sheet 2:** Clinical and laboratory characteristics of samples and individuals of the longitudinal cohort (Cohort 1). **Sheet 3:** Characteristics of samples and individuals included in the seasonal analysis (Cohort 1) and the corresponding validation analysis (Cohort 3). **Sheet 4:** Characteristics of samples and individuals included in the analysis of sex-specific gene expression variation (Cohort 1) and the corresponding validation analysis (Cohort 3).

File Name: Supplementary Data 2

Description: Summary of gene-level findings resulting from the study. Sheet 1: Data dictionary. **Sheet 2:** Summary table.

File Name: Supplementary Data 3

Description: Co-expression module characteristics and association with clinical parameters. Sheet 1: Data dictionary. **Sheet 2:** Associations between module eigengenes and clinical parameters. Associations were assessed using linear mixed-effects models (see Methods). Significance of associations was determined by comparing the full to a reduced model and resulting p-values were adjusted for multiple testing using the Benjamini and Hochberg method.
