# Supplementary material for: Large-scale analysis of temporal gene expression variation in peripheral blood
Source: Nat Commun. 2026 May 29;17:6992. doi: 10.1038/s41467-026-73218-6 (PMC13392353; doi:10.1038/s41467-026-73218-6)
Supplement: Supplementary file 6 — Reporting Summary [file 41467_2026_73218_MOESM6_ESM.pdf]

nature portfolio

Corresponding author(s): Philip Rosenstiel, Jeroen Raes

Last updated by author(s): 30.04.2026

# Reporting Summary

Nature Portfolio wishes to improve the reproducibility of the work that we publish. This form provides structure for consistency and transparency in reporting. For further information on Nature Portfolio policies, see our Editorial Policies and the Editorial Policy Checklist.

## Statistics

For all statistical analyses, confirm that the following items are present in the figure legend, table legend, main text, or Methods section.

| n/a | Confirmed | |
|---|---|---|
| [ ] | [x] | The exact sample size (*n*) for each experimental group/condition, given as a discrete number and unit of measurement |
| [ ] | [x] | A statement on whether measurements were taken from distinct samples or whether the same sample was measured repeatedly |
| [ ] | [x] | The statistical test(s) used AND whether they are one- or two-sided<br>*Only common tests should be described solely by name; describe more complex techniques in the Methods section.* |
| [ ] | [x] | A description of all covariates tested |
| [ ] | [x] | A description of any assumptions or corrections, such as tests of normality and adjustment for multiple comparisons |
| [ ] | [x] | A full description of the statistical parameters including central tendency (e.g. means) or other basic estimates (e.g. regression coefficient) AND variation (e.g. standard deviation) or associated estimates of uncertainty (e.g. confidence intervals) |
| [ ] | [x] | For null hypothesis testing, the test statistic (e.g. *F*, *t*, *r*) with confidence intervals, effect sizes, degrees of freedom and *P* value noted<br>*Give P values as exact values whenever suitable.* |
| [x] | [ ] | For Bayesian analysis, information on the choice of priors and Markov chain Monte Carlo settings |
| [ ] | [x] | For hierarchical and complex designs, identification of the appropriate level for tests and full reporting of outcomes |
| [ ] | [x] | Estimates of effect sizes (e.g. Cohen's *d*, Pearson's *r*), indicating how they were calculated |

*Our web collection on statistics for biologists contains articles on many of the points above.*

## Software and code

Policy information about availability of computer code

| | |
|---|---|
| Data collection | Sequencing Base calling: bcl2fastq v2.20 |
| Data analysis | RNA-seq data analysis: nf-core rnaseq pipeline v1.3, Trim Galore v0.5.0, STAR aligner v2.6.1d, featureCounts v1.6.4, mixOmics v6.24.0, variancePartition v1.30.2, StringTie v1.3.5, tximport v1.28.0, DRIMSeq v1.28.0, RUVSeq v1.28.0<br>Cell-type deconvolution: CIBERSORTx<br>Intraclass correlation analysis: ICC v2.4.0, lme4 v1.1-37<br>Heritability analysis: STAR aligner v2.6.1a, QTLtools v1.3.1, GenABEL v1.8-0, lme4 v1.1-37, mets<br>Seasonal analysis: Maaslin2 v1.14.1<br>Functional enrichment analysis: topGO v2.46.0<br>Gene co-expression analysis: WGCNA v1.72, nlme v3.1-162<br>Rhineland cohort analysis: FastQC v0.11.9, STAR aligner v2.7.1<br>Statistical analysis: R v4.1.1 for heritability analysis, R v4.3.1 for all remaining analyses<br>Custom code: https://github.com/Systems-Immunology-IKMB/Temporal_gene_expression_variation.<br>Genome version: GRCh38 (GENCODE v25) |

For manuscripts utilizing custom algorithms or software that are central to the research but not yet described in published literature, software must be made available to editors and reviewers. We strongly encourage code deposition in a community repository (e.g. GitHub). See the Nature Portfolio guidelines for submitting code & software for further information.

## Data

Policy information about availability of data

All manuscripts must include a data availability statement. This statement should provide the following information, where applicable:

- Accession codes, unique identifiers, or web links for publicly available datasets
- A description of any restrictions on data availability
- For clinical datasets or third party data, please ensure that the statement adheres to our policy

RNA-seq data generated for Cohort 1 (333 individuals) have been deposited in the European Genome-Phenome Archive with accession code EGAD50000000859. To protect the privacy of the individuals, these datasets including processed files and metadata are available under restricted access. Requests for access can be submitted directly in EGA. These requests are reviewed by the VIB Data Access Committee within a reasonable timeframe. Data can be shared for research purposes that are compatible with the original goal for which the data were collected. Prior to the sharing of any data a Data Sharing Agreement with VIB must be completed that will include the necessary conditions to guarantee the protection of personal data and limit secondary use to a specified research activity.
All data relating to TwinsUK samples, which Cohort 2 is a subset of, have been deposited to the TwinsUK BioResource data management team. These data are available by application to the Twin Research Executive Access committee (TREC) at King's College London. The TwinsUK BioResource is managed by TREC, which provides governance of access to TwinsUK data and samples to researchers investigating health, well-being or disease. TwinsUK data users are bound by data sharing agreement set out in the data access application form, which includes responsibilities with respect to third party data sharing and maintaining participant privacy. Further responsibilities include a responsibility to acknowledge data sharing. Please see https://twinsuk.ac.uk/researchers/access-data-and-samples/request-access/ for additional information and to access data access proposal forms.
The data from the Rhineland Study (Cohort 3) used in this manuscript are not publicly available due to data protection and privacy regulations. For the Rhineland Study, access can be obtained by submitting a formal request to the Data Access Committee (RS-DUAC) for academic, non-commercial research purposes, and in accordance with the Rhineland Study's Data Use and Access Policy. The committee will review all requests within 4 weeks. Access may be subjected to a completed data transfer agreement. Requests for further information or to access the Rhineland Study's dataset should be directed to RS-DUAC@dzne.de.
Source data are provided with this paper.

## Research involving human participants, their data, or biological material

Policy information about studies with human participants or human data. See also policy information about sex, gender (identity/presentation), and sexual orientation and race, ethnicity and racism.

| | |
|---|---|
| Reporting on sex and gender | We have reported biological sex of all human subjects involved. Sex was considered in the study design and was determined based on self-reporting. Sex-segregated gene expression analysis was performed, and sex-segregated cohort information has been provided in Supplementary Table 1. Individual-level data cannot be shared publicly due to data protection laws but can be obtained under restricted access from the EGA submission. The longitudinal study cohort (Cohort 1) consisted of 220 female and 113 male participants while the Rhineland cohort (Cohort 3) consisted of 1,607 female and 1,335 male participants. |
| Reporting on race, ethnicity, or other socially relevant groupings | Race, ethnicity, or other socially relevant groupings were not considered in the analysis. |
| Population characteristics | Participants of the longitudinal study cohort (Cohort 1) resided in Belgium at the time of recruitment and were selected to reflect a Western European population. As such participant characteristics represent a diverse range of age (median 48 years [19-70]), sex (66.1% female), health conditions such as chronic inflammatory diseases (12.3%), depression (11.4%) and hay fever (17.7%), immunological events including acute infections at the timepoint of sampling (4.9%), and other lifestyle factors.<br>The MultiMuther study (Cohort 2) consists of European participants from the UK, including 148 monozygotic twins and 166 dizygotic twins and 21 singletons.<br>The Rhineland Study (Cohort 3) is an on-going population-based cohort study consisting of individuals living in Bonn, Germany. Participants of the study are 30 years of age or older. |
| Recruitment | Participants of Cohort 1 were recruited from volunteers of the Flemish Gut Flora Project, a larger population study, who expressed interest in participating in follow-up studies, and the general population. Additional volunteers were recruited through online advertising.<br>For Cohort 3 invitations to join the study were extended to all residents of two geographically defined areas of Bonn who are 30 years of age or older and have sufficient German language proficiency to provide informed consent. |
| Ethics oversight | All procedures and protocols regarding Cohort 1 were approved by the Ethics Committee Research of University Hospitals Leuven (Belgium) (study ID S60030) and align with the Declaration of Helsinki and Belgian privacy laws.<br>TwinsUK, which Cohort 2 is a subset of, main ethics was reviewed and approved by the NHS London—London Bridge Research Ethics Committee (REC reference EC/04/015) and by Guy's and St Thomas' NHS Foundation Trust Research and Development (R&D) in 2012. TwinsUK BioBank was approved by NHS North West—Liverpool East Research Ethics Committee (REC reference 19/NW/0187), IRAS ID 258513. All research was therefore carried out in accordance with the ethical standards laid down in the 1964 Declaration.<br>The Rhineland study (Cohort 3) received approval from the ethics committee of the Medical Faculty at the University of Bonn (reference ID: 338/15). It is conducted in compliance with the International Conference on Harmonization (ICH) Good Clinical Practice (GCP) standards (ICH-GCP). Written informed consent was obtained from all participants in accordance with the Declaration of Helsinki. |

Note that full information on the approval of the study protocol must also be provided in the manuscript.

# Field-specific reporting

Please select the one below that is the best fit for your research. If you are not sure, read the appropriate sections before making your selection.

- [x] Life sciences
- [ ] Behavioural & social sciences
- [ ] Ecological, evolutionary & environmental sciences

For a reference copy of the document with all sections, see nature.com/documents/nr-reporting-summary-flat.pdf

# Life sciences study design

All studies must disclose on these points even when the disclosure is negative.

| | |
|---|---|
| Sample size | No formal sample-size calculation was performed. All samples from individuals, that had blood samples at all three time points available, were used (334 individuals, 1002 samples). |
| Data exclusions | Data from one participant (3 samples) was excluded from the analysis due to diagnosis of cancer and ongoing treatment. Additionally, samples that failed sequencing or pre-processing quality control were excluded from the analysis (29 samples). |
| Replication | We have replicated our results on sex-specific differences in inter-individual expression variation and seasonal transcriptional patterns using RNA-Seq data from the Rhineland cohort, an external cross-sectional cohort of 3,480 individuals. Using this dataset, we validated higher inter-individual expression variation in male compared to female participants and showed that this pattern persisted after stratifying for menopausal status similarly to the longitudinal cohort. Moreover, we confirmed seasonal expression patterns of several biological processes, including the upregulation of IL-12 production, response to IL-7, sphingosine-1 phosphate receptor signaling, and chromatin remodeling in winter and the upregulation of multiple circadian rhythm genes and genes involved in Notch signaling in summer. Replication of results related to the longitudinal nature of the study including findings from the variance partition analysis, intraclass correlation analysis, eQTL analysis, and "outlier gene" analysis was not possible due to the lack of longitudinal studies with a similar experimental setup and patient characteristics. |
| Randomization | Randomization was irrelevant as no experimental groups were assigned. |
| Blinding | Blinding was irrelevant because no group allocation was performed. |

# Reporting for specific materials, systems and methods

We require information from authors about some types of materials, experimental systems and methods used in many studies. Here, indicate whether each material, system or method listed is relevant to your study. If you are not sure if a list item applies to your research, read the appropriate section before selecting a response.

## Materials & experimental systems

| n/a | Involved in the study | |
|---|---|---|
| [x] | [ ] | Antibodies |
| [x] | [ ] | Eukaryotic cell lines |
| [x] | [ ] | Palaeontology and archaeology |
| [x] | [ ] | Animals and other organisms |
| [x] | [ ] | Clinical data |
| [x] | [ ] | Dual use research of concern |
| [x] | [ ] | Plants |

## Methods

| n/a | Involved in the study | |
|---|---|---|
| [x] | [ ] | ChIP-seq |
| [x] | [ ] | Flow cytometry |
| [x] | [ ] | MRI-based neuroimaging |

## Plants

| | |
|---|---|
| Seed stocks | *Report on the source of all seed stocks or other plant material used. If applicable, state the seed stock centre and catalogue number. If plant specimens were collected from the field, describe the collection location, date and sampling procedures.* |
| Novel plant genotypes | *Describe the methods by which all novel plant genotypes were produced. This includes those generated by transgenic approaches, gene editing, chemical/radiation-based mutagenesis and hybridization. For transgenic lines, describe the transformation method, the number of independent lines analyzed and the generation upon which experiments were performed. For gene-edited lines, describe the editor used, the endogenous sequence targeted for editing, the targeting guide RNA sequence (if applicable) and how the editor was applied.* |
| Authentication | *Describe any authentication procedures for each seed stock used or novel genotype generated. Describe any experiments used to assess the effect of a mutation and, where applicable, how potential secondary effects (e.g. second site T-DNA insertions, mosiacism, off-target gene editing) were examined.* |
