# Supplementary material for: Large-scale analysis of temporal gene expression variation in peripheral blood
Source: Nat Commun. 2026 May 29;17:6992. doi: 10.1038/s41467-026-73218-6 (PMC13392353; doi:10.1038/s41467-026-73218-6)
Supplement: Supplementary file 21 — Transparent Peer Review file [file 41467_2026_73218_MOESM21_ESM.pdf]

nature portfolio

Peer Review File

Large-scale analysis of temporal gene expression variation in peripheral blood

Corresponding Author: Professor Philip Rosenstiel

Parts of this Peer Review File have been redacted as indicated to maintain the confidentiality of unpublished data.

This file contains all reviewer reports in order by version, followed by all author rebuttals in order by version.

A version of this paper was originally rejected for publication by Nature Communications, however that decision was reconsidered after appeal by the authors.

Version 0:

Reviewer comments:

Reviewer #1

(Remarks to the Author)
The manuscript presents a longitudinal transcriptomic dataset with valuable insights into intra- vs inter-individual variability, splicing dynamics, sex- and season-specific effects, and clinical implications. The authors conclude that intra-individual gene expression variations is larger than inter-individual expression variation, with ramifications when people want to identify reliable gene expression based biomarkers.

Major comments:

The authors conclude that intra-individual variation in blood gene expression exceeds inter-individual variation for most genes. While the analysis is rigorous, I would encourage the authors to frame this finding more in line with what is already known from eQTL studies. It has long been observed that systematic inter-individual differences explain only a modest fraction of total variance, with genetic factors accounting for a relatively small proportion of expression variability. Similarly, eQTL discovery rates typically substantially increase after correction for expression principal components, underscoring that a large component of variance reflects dynamic, non-genetic factors. In this context, it is perhaps less surprising that within-individual temporal fluctuations outweigh mean differences between individuals, particularly in a genetically and environmentally homogeneous cohort. I suggest that the authors discuss their findings with reference to this prior literature, and clarify what new insights are gained here—for example, the identification of specific biological processes or gene sets that are more intra- versus inter-individually variable, or the implications for biomarker reproducibility in clinical studies.

I noticed some earlier studies that seem to be be addressing related questions and that have not been cited (the authors only cite earlier repeated longitudinal expression quantification work by Whitney et al, PNAS 2003):
- Rose et al, Nature Medicine 2019 also studied longitudinal gene expression in a substantial number of individuals. The authors do not refer to this paper, nor do they compare their findings with the findings of that study. What are the key differences in results from this manuscript, as compared to Rose et al?
- Rondina et al, Circ Res 2020 also studied longitudinal gene expression in platelets, and "examined within and between individual variation and repeatability of platelet RNA expression and exon skipping". What are the key differences in results from this manuscript, as compared to Rondina et al?

The authors do not indicate that they used cell-type decomposition approaches to check whether cell-type composition differences might explain the differences. Can the authors comment on this? I believe it would be important to check this.

Minor comments:

I would like to get an idea of how the authors think the field should move forward in order to identify reliable gene expression based biomarkers. I would be inclined to think that single-cell RNA-seq might be useful, since it will account for (subtle) cell-type composition differences that is a cause of expression variation in bulk data. Can the authors provide guidelines in the

discussion section on this?

(Remarks on code availability)

Reviewer #2

(Remarks to the Author)
This manuscript presents an interesting longitudinal study of peripheral blood RNA sequencing. The results of the analysis on inter vs intra individual variation, seasonal variation and expression modules are notable and informative for future studies. This may also be a useful data resource for further analyses. I do have a few concerns I would like to see addressed as well as some general comments and questions.

Concerns:
- My primary concern in this manuscript is regarding the use of methods that generally assume sample independence on longitudinal data. Specifically - the use of PCA to assess variance contribution, the use of RUVg to remove batch effects, and the use of WGCNA to find modules. While one can obviously apply PCA to any matrix, repeated measures or not, that doesn't necessarily mean this is correct. Standard interpretations of PCA implicitly assume sample independence which is violated here. A better approach would be a factor model that takes the longitudinal nature of the data into account. See An et al Stat Med 2013 for one possibility, there may be others.
- Relatedly, the methods do not make clear whether RUVg was applied to each timepoint, or all data together. This should be clarified. As RUVg is effectively just performing PCA on a set of a priori non-differentially-expressed genes followed by regression, it is also invalid to apply this to longitudinal data all together. Unfortunately I am not aware of what best practices for batch correction for longitudinal RNA-seq data is. One could apply RUVg or similar to each timepoint, but this may not completely correct the effects. In principle one could apply the above referenced method to the reference gene set and follow a similar procedure to RUVg.
- We have the same issue for WGCNA. The base calculation is a standard correlation between expression levels, which is not accounting for the repeated measures. Again one could use a mixed model to estimate the correlation, but I am not sure how amenable the WGCNA software would be to supplying this modified correlation matrix. Notably, this does present an interesting opportunity to study the stability of identified WGCNA modules across time that hasn't been explored. If you run WGCNA on each timepoint do you identify the same modules? If you run WGCNA on a single timepoint, which modules are stable across time and which aren't?
- I am a bit concerned around the framing of the results of non-protein coding genes. Given the mRNA sampling protocol, this isn't exactly an unbiased sample of the non-coding transcriptome. I realize this is mentioned briefly in the discussion, however I am not sure it is fair to frame the results as characterizing properties of both coding and non-coding RNA when only ~1/5th of them are represented.
- The methods write up around the analysis of infections is difficult to follow and it is unclear what was done. You have individuals with infections at some time points, and then you have matched controls, which makes sense. But then the samples from these same individuals during non-infectious timepoints are also used and it isn't clear to me how this is done. How are repeated measures handled here?

Other comments/questions:
- The claim on paige 8 that heritable genes with higher inter-individual variability suggest environmental factors seems a bit strong given that there are several environmental factors measured here and most variance still seems to be in the residuals, suggesting random noise or inadequate power. Could an analysis be done to further support this claim?
- The analysis corresponding to Fig 6 is, to me, perhaps the most interesting result here. Is this related to so-called "frequent DEGs" that come up across many studies? Are these more or less heritable than the others? Could this variation be impacting eQTL studies as well?
- If possible, I would strongly prefer to see anonymized transcript quantification results uploaded to a data sharing platform such as Zenoodo for FigShare, rather than raw reads uploaded to a read archive available on "reasonable request". This would enable reproduction of the results and reduce the barrier for further analyses.

Minor comments:
- The text in every figure is too small
- I'm unclear on the difference between Figure 1c and ED2b

(Remarks on code availability)
Scripts corresponding to major analyses seem to be present in the repository. As the data are only available on reasonable request, replication of the results or testing the code is not straightforward.

Version 1:

Reviewer comments:

Reviewer #1

(Remarks to the Author)
The authors have largely addressed my main concerns in a technically satisfactory way. The manuscript is now clearer, better contextualized, and more rigorous:
- The authors addressed the concern regarding cellular heterogeneity by incorporating both measured blood counts and detailed deconvolution analyses, demonstrating that the main findings are robust to cell-type composition effects.
- The authors now integrate their findings with eQTL literature in the Discussion, acknowledging that genetic effects explain only a modest fraction of expression variance and positioning their results within that framework.
- The authors now refer to previous papers that studied the same phenomena but at a smaller scale.

(Remarks on code availability)

Reviewer #2

(Remarks to the Author)
I appreciate the thorough response to my original comments. I particularly appreciate the re-analysis of the temporal RNA-seq using mixOmics and modification of some downstream analyses to used mixed models. The new analysis of frequently DEG and heritability is very interesting, and the text clarifications are helpful.

Regarding the use of RUVg and WGCNA, I understand the counter-points the authors are making. As a meta-comment, I am frequently a bit frustrated by the way longitudinal data is(n't) handled in genomics research. On the methods side, the community needs to do a better job creating and promoting methods that can handle these data rigorously; and analysts need to actually use them. It is certainly not my goal to ask the authors of this manuscript to solve this entire field here!

I appreciate the additional citations and discussion and agree these uses are in line with current best practices, although I might contend there are some issues with current best practices, that is outside the scope of this paper. I agree that the proposed alternative ways of constructing WGCNA modules would likely attenuate signal, but I am still concerned that this use may bias the module identification to find things that are a bit more individual specific. However I will concede the results still seem biologically meaningful and are corroborated by the individual time-point analysis done for the review, so this may not be a large issue in practice. In any case, I agree that further explorations of this topic are (well) beyond the scope of this manuscript and do not intend to further belabor the point.

(Remarks on code availability)
Results cannot be verified due to data usage restrictions that the authors have stated were required.

Open Access This Peer Review File is licensed under a Creative Commons Attribution 4.0 International License, which permits use, sharing, adaptation, distribution and reproduction in any medium or format, as long as you give appropriate credit to the original author(s) and the source, provide a link to the Creative Commons license, and indicate if changes were made.
In cases where reviewers are anonymous, credit should be given to 'Anonymous Referee' and the source.
The images or other third party material in this Peer Review File are included in the article’s Creative Commons license,

unless indicated otherwise in a credit line to the material. If material is not included in the article’s Creative Commons license and your intended use is not permitted by statutory regulation or exceeds the permitted use, you will need to obtain permission directly from the copyright holder.
To view a copy of this license, visit https://creativecommons.org/licenses/by/4.0/

Reviewer #1 (Remarks to the Author):

The manuscript presents a longitudinal transcriptomic dataset with valuable insights into intra- vs inter-individual variability, splicing dynamics, sex- and season-specific effects, and clinical implications. The authors conclude that intra-individual expression variations is larger than inter-individual expression variation, with ramifications when people want to identify reliable expression based biomarkers.

Major comments:

The authors conclude that intra-individual variation in blood expression exceeds inter-individual variation for most genes. While the analysis is rigorous, I would encourage the authors to frame this finding more in line with what is already known from eQTL studies. It has long been observed that systematic inter-individual differences explain only a modest fraction of total variance, with factors accounting for a relatively small proportion of expression variability. Similarly, eQTL discovery rates typically substantially increase after correction for expression principal components, underscoring that a large component of variance reflects dynamic, non-genetic factors. In this context, it is perhaps less surprising that within-individual temporal fluctuations outweigh mean differences between individuals, particularly in a genetically and environmentally homogeneous cohort. I suggest that the authors discuss their findings with reference to this prior literature, and clarify what new insights are gained here—for example, the identification of specific biological processes orsets that are more intra- versus inter-individually variable, or the implications for biomarker reproducibility in clinical studies.

We thank the reviewer for this insightful comment and agree that our findings should be contextualized with what is already known from eQTL studies. Indeed, genetic differences between individuals are known to explain only a small percentage of expression variation, as shown by heritability studies (Wright et al., 2014, Nature Genetics) and eQTL rates can generally be improved by correcting for expression principal components (Stegle et al., 2012, Nature Protocols), highlighting the importance of dynamic, non-genetic variability.

In essence, our study extends the eQTL studies in five ways:
i. Our study provides direct partitioning of inter- and intra-individual variance by quantifying for each gene the proportion of variance due to between-individual and within-individual temporal fluctuation.
ii. We quantify the contribution of a multitude of environmental, technical and phenotypical factors to overall transcriptional variance per gene.
iii. We systematically identify biological processes and gene sets that are preferentially variable between individuals (eg. genes from the HLA locus, T cell receptor-associated genes and genes involved in cell adhesion, chemokine-mediated signalling pathway) versus within individual (eg. Ribosomal, genes related to splicing and translation), clarifying which pathways may be unstable over time and therefore less suitable for static biomarker development.
iv. We demonstrate that inter- and intra-individual variance differs between males and females and identify specific gene sets and processes exhibiting sex-dependent variability patterns.
v. We identify a set of transcripts with potentially overestimated inter-individual variation in cross-sectional studies, which should be evaluated cautiously as some differences may also arise from physiological variability.

We have revised both the Introduction (lines 94-101) and Discussion (lines 512-522, 525-534) and cited additional studies (lines 95, 101, 518) to explicitly integrate prior knowledge from eQTL studies and highlight new insights provided by our study.

I noticed some earlier studies that seem to be be addressing related questions and that have not been cited (the authors only cite earlier repeated longitudinal expression quantification work by Whitney et al, PNAS 2003):
- Rose et al, Nature Medicine 2019 also studied longitudinal expression in a substantial number of individuals. The authors do not refer to this paper, nor do they compare their findings with the findings of that study. What are the key differences in results from this manuscript, as compared to Rose et al?

We thank the reviewer for highlighting this relevant work. While both studies investigate longitudinal patterns, the two studies differ in scientific objectives, study populations, and analytical frameworks.

- Rose et al. performed intensive longitudinal multi-omics profiling of 109 individuals at risk for type II diabetes mellitus to examine personalized trajectories into chronic disease and evaluate disease prediction through different Omics measures. They used gene expression data from isolated PBMCs from 51 samples primarily as one component of a multi-layer prediction framework and to compute composite disease scores based on known risk genes.
- In contrast, our study was specifically designed to characterize natural gene expression variability in a population cohort. Rather than studying disease onset, we directly quantify and partition inter- and intra-individual variability and investigate different sources of variation such as sex, seasonal effects, and splicing events, and evaluate how these sources of variability affect biomarker discovery with recommendations for cross-sectional studies. Consequently, we have performed RNA sequencing on whole blood as opposed to PBMCs.

Overall, the two studies reveal complementary insights: Rose et al. demonstrate large disease-related multi-omic perturbations, whereas our work maps the natural variability landscape of the whole-blood transcriptome. **We have now cited Rose et al. (Nature Medicine, 2019) in the Introduction (lines 88-91) and Discussion (lines 481-485) to contextualize our findings.**

Note: We also attempted to access the longitudinal transcriptome data from Rose et al. to further validate our findings and potentially perform a direct comparison of variance components. However, the data availability link provided in the manuscript is currently not functional, and we were unable to locate the dataset through the indicated repository.

- Rondina et al, Circ Res 2020 also studied longitudinal expression in platelets, and "examined within and between individual variation and repeatability of platelet expression and exon skipping". What are the key differences in results from this manuscript, as compared to Rondina et al?

We thank the reviewer for pointing out this valuable study by Rondina et al. and comment on differences in (1) study design and repeatability results and (2) analytical focus below.

(1) Although both analyses investigate within- and between-individual transcriptional variation, the studies differ substantially in tissue type, sampling design, analytical focus, and biological questions addressed. Rondina et al. profiled platelet transcriptome in two small cohorts (Cohort 1, n=31, T0, 4 months; Cohort 2, n=7, T0, 2 weeks, 1 year, 4 years) to assess repeatability and improve QTL detection, whereas our study analyses whole-blood RNA-seq from a larger population-based cohort (n=333, T0, 3 months, 6 months). These differences in tissue composition, cohort size, and sampling frequency likely explain the contrasting global variance patterns. Rondina et al. reported highly stable platelet expression (>50% inter-individual variance), while we observe substantially greater within-individual temporal variability in whole blood (85% of genes with greater intra-individual variation).

To investigate differences in repeatability results, we performed a direct comparison of the repeatability patterns across studies (see Figure 1 below). Adaptive immune genes showed consistent behaviour across both studies, with high ICC in whole blood and high repeatability in both platelet cohorts. In contrast, genes related to cell cycle, transcription, translation and splicing showed divergent patterns between studies and even between Rondina et al.'s two cohorts. These comparisons show that gene expression variability is cell type dependent and sensitive to time scale of sampling. This comparison was performed to address the reviewer's question. However, given the substantial differences in tissue type, study design, and analytical approaches, **we did not include the full analysis in the manuscript. Instead, we added a brief statement in the Discussion (lines 518-520) highlighting the key similarities and differences between our findings and those of Rondina et al.**

editorial note: unpublished, confidential information redacted]

(2) While Rondina et al. focus on improving genetic signal detection (eQTL and sQTL mapping), our study aims to characterize the landscape of natural transcriptional variability and evaluate its consequences for study design and biomarker reproducibility in clinical settings. Motivated by the parallels with their work, we have now extended our analyses to explicitly examine how inter- and intra-individual variation influences eQTL detection in whole blood, and **we present these new results regarding eQTL discovery in Section "Impact of intra-individual variability on the accuracy of cohort-based transcriptomic studies" (lines 423-440), Figures 6a-d and Extended Data Figures 9a-c and have edited the Methods (lines 934-949) and Discussion (lines 516-522) to include this analysis.**

The authors do not indicate that they used cell-type decomposition approaches to check whether cell-type composition differences might explain the differences. Can the authors comment on this? I believe it would be important to check this.

We agree with the reviewer that accounting for cell-type composition is important.

In the original manuscript, we had already incorporated laboratory-measured blood cell counts, which included neutrophils, lymphocytes, monocytes, eosinophils, and thrombocytes, for all samples directly into our variance partitioning framework (Figure 1) and additionally quantified the ICC of cell-type abundances, which was reported in the Results. Specifically, most cell types exhibited greater inter-individual than intra-individual variability, with neutrophils being a notable exception, where approximately 55% of the variance arose from within-individual fluctuations (Extended Data Fig. 3c). These results indicate that cell-type composition can explain only part of the observed intra-individual variability in gene expression.

In response to the reviewer's comment, we have now extended our analysis by estimating detailed cell-type composition using CIBERSORTx, including 22 major leukocyte populations such as naïve and memory B cells, plasma cells, CD8+ T cells, naïve and memory resting and activated CD4+ T cells, follicular helper T cells, regulatory T cells, gd T cells, resting and activated NK cells, monocytes, macrophages (M0, M1, M2), resting and activated dendritic cells, resting and activated mast cells, eosinophils, and neutrophils, and recalculating gene-level ICCs after adjusting for inferred cell proportions. While both inter- and intra-individual variance components were reduced after this adjustment, the overall ICC distribution changed only marginally, and adjusted and unadjusted ICCs were highly correlated. This demonstrates that cell-type composition affects both variance components similarly and does not account for the predominance of intra-individual transcriptional variability observed in our data. **These results on cell-type adjusted ICC have been included in the result section "Intra-individual gene expression variability surpasses inter-individual differences" (lines 212-220), Figures 2b-c, Extended Data Fig. 3d. The methodology is included in the Methods (lines 708-725), and the findings are discussed in the Discussion (lines 499-507).**

Minor comments:

I would like to get an idea of how the authors think the field should move forward in order to identify reliable expression based biomarkers. I would be inclined to think that single-cell RNA-seq might be useful, since it will account for (subtle) cell-type composition differences that is a cause of expression variation in bulk data. Can the authors provide guidelines in the discussion section on this?

We thank the reviewer for this constructive suggestion and agree that clearer guidance for different gene expression analyses would strengthen the manuscript.

Specifically, we propose the following recommendations:

- Genes/processes with high inter-individual and low intra-individual variance (high ICC) should be prioritized for eQTL discovery. Based on our analysis, we suggest an ICC threshold of ~0.5 for trans-eQTL analysis and single cohort cis-eQTL analysis and ~0.25 for multi-cohort cis-eQTL analysis.
- Test-retest designs and cross-cohort validations should be incorporated for biomarker discovery and temporal reliability metrics should be reported to assess generalizability and stability of candidate biomarkers.
- The effect of annual season should be considered for cross-sectional cohorts that sample individuals across different times of the year.
- Sex and other major sources of technical, environmental, and biological variation should be quantified and modelled, and the significant contributors should be included as covariates in the downstream analysis.
- Since cell type composition can inflate both inter- and intra-individual differences in bulk RNA-seq data, it should be explicitly considered. This can be addressed by modelling measured or inferred cell type proportions. While single-cell RNA-seq can help disentangle the cell type specific effects, some biomarkers may reflect composite signals across multiple cell types and may therefore be more robustly detected and validated in bulk data.

We have included these practical recommendations in the Discussion (lines 616-627).

Reviewer #2 (Remarks to the Author):

This manuscript presents an interesting longitudinal study of peripheral blood sequencing. The results of the analysis on inter vs intra individual variation, seasonal variation and expression modules are notable and informative for future studies. This may also be a useful data resource for further analyses. I do have a few concerns I would like to see addressed as well as some general comments and questions.

Concerns:
- My primary concern in this manuscript is regarding the use of methods that generally assume sample independence on longitudinal data. Specifically - the use of PCA to assess variance contribution, the use of RUVg to remove batch effects, and the use of WGCNA to find modules. While one can obviously apply PCA to any matrix, repeated measures or not, that doesn't necessarily mean this is correct. Standard interpretations of PCA implicitly assume sample independence which is violated here. A better approach would be a factor model that takes the longitudinal nature of the data into account. See An et al Stat Med 2013 for one possibility, there may be others.

We agree that standard implementations of these methods typically assume independence and may lead to biased inference in the presence of repeated measures; **we have therefore re-analyzed the data using models appropriate for longitudinal designs where applicable and have further clarified how we used these tools in the Methods.**

For dimensionality reduction, we did not find an implementation of the suggested algorithm by An et al. that had already been tested on data comparable to ours. Instead, we have now used a multilevel approach designed for sequencing data with repeated measures or longitudinal design that was proposed by Liquet et al. (BMC Bioinformatics, 2012). Briefly, the expression counts are decomposed into within- and between-subject matrices, on which PCA can then be applied separately. This allowed us to investigate drivers of inter- and intra-individual variation of the global expression profiles while taking sample dependence into account. It is important to note that the contribution of investigated factors to inter-individual variation is comparable to our previous results obtained with standard PCA so that our main conclusions did not change by applying the alternative method. **We have included the results of this analysis in the Result section "Sample donor and cellular blood composition are major sources of variation in gene-level expression data" (lines 149-161), Methods (lines 674-682), and added both the inter- and intra-individual variation factor plots and correlation between both factor groups and metadata in Figures 1a-d and Extended data Fig. 2a and removed the initial PCA analysis.**

- Relatedly, the methods do not make clear whether RUVg was applied to each timepoint, or all data together. This should be clarified. As RUVg is effectively just performing PCA on a set of a priori non-differentially-expressed genes followed by regression, it is also invalid to apply this to longitudinal data all together. Unfortunately I am not aware of what best practices for batch correction for longitudinal RNA-seq data is. One could apply RUVg or similar to each timepoint, but this may not completely correct the effects. In principle one could apply the above referenced method to the referenceset and follow a similar procedure to RUVg.

We appreciate the concern that sample dependence violates the assumptions of the method. Risso et al. introduced RUVg in Nature Biotechnology 2014 and tested the method on an RNA-sequencing data set from the Sequencing Quality Control (SEQC) Consortium which contains technical replicates at both the library preparation and sequencing level and external qRT-PCR controls for two RNA samples, sample A and sample B. They found that *"RUVg effectively reduced library preparation effects for the SEQC data set without weakening the sample A versus B effect"* indicating that RUVg provides reasonable results even for repeated measures. Risso et al. additionally state assumptions of the RUV approaches and comment on the special case of time-course differential expression settings. While Risso et al. discuss that certain assumptions of RUVg may be challenged in time-course differential expression analyses when using empirical controls, they do not report concerns regarding the use of negative control genes in longitudinal settings.

However, we recognize that every normalization method must be evaluated carefully and we rigorously assessed the impact of RUVg in our data. After RUVg factor identification, we conducted variance partition analysis on gene expression counts adjusted for each factor separately (Extended Data Fig. 2c). We selected factors that substantially reduced technical variance associated with sequencing run and cohort, while preserving variance attributable to major biological factors such as donor identity and neutrophil abundance. These results indicate that RUVg effectively captured technical variation without attenuating biologically

meaningful inter- and intra-individual signals in our longitudinal dataset. **We have included this explanation in the Methods (lines 739-744) and discussed the limitations of the method in the Discussion (lines 591-598). Additionally, we have clarified in the Methods that RUVg was used on all samples together (lines 744-746).**

- We have the same issue for WGCNA. The base calculation is a standard correlation between expression levels, which is not accounting for the repeated measures. Again one could use a mixed model to estimate the correlation, but I am not sure how amenable the WGCNA software would be to supplying this modified correlation matrix. Notably, this does present an interesting opportunity to study the stability of identified WGCNA modules across time that hasn't been explored. If you run WGCNA on each timepoint do you identify the same modules? If you run WGCNA on a single timepoint, which modules are stable across time and which aren't?

We agree that assessing the stability of co-expression modules across individual timepoints represents an interesting and important methodological extension and value the reviewer's concern regarding the use of standard correlation-based WGCNA in a longitudinal setting. We address these two points separately below.

(1) As the reviewer suggested, we have taken this opportunity to test the preservation of co-expression modules over time by constructing separate sets of modules for each season and performing module preservation analysis (see Figure 2 below). For comparability, we have focused on individuals from Cohort A with samples available at all three sampling timepoints (242 individuals). We identified 29, 28, 24 modules from winter-, spring-, and summer-specific networks, respectively, with all modules showing moderate to high preservation in the respective other seasons based on the Zsummary score (median [min, max] 9.1 [2.4, 39.5]). As the Zsummary score predominantly correlated with module size, we focused on the medianRank to identify the least preserved modules for each season. medianRank values were highly correlated between seasons and the least preserved modules based on this measure were predominantly enriched in different immune related processes, including defense response to virus and platelet aggregation and activation.

editorial note: unpublished, confidential information redacted]

We feel that a more extensive analysis would be needed to fully address this question, which would be beyond the scope of the current study. As we already report on seasonal effects and temporal changes in viral response processes in separate analyses (Sections "Seasonal variations significantly influence gene expression and transcript usage" and "Associations between gene co-expression modules, clinical parameters, and common medical conditions"), **we have not included results of the preservation analysis in the manuscript.**

(2) Regarding the use of WGCNA in a longitudinal setting we would like to note that in our study, WGCNA was used as an exploratory, hypothesis-generating tool rather than as a formal statistical test, and despite the violation of strict independence assumptions, the identified modules were biologically relevant. In particular, the co-expression modules highlighted infection- and allergy-associated signatures, underscoring the utility of the cohort for discovering meaningful longitudinal transcriptional patterns.

We also discussed this issue directly with Peter Langfelder, one of the authors of the WGCNA package, who agreed that our analytical approach is appropriate for the exploratory aims of this study. Importantly, our scientific question focuses on the temporal modulation of module eigengenes. Alternative strategies, such as constructing networks from per-subject means, residuals from mixed-effects models, or decorrelated expression matrices, risk removing or attenuating the very temporal variation that drives co-expression in longitudinal data and is central to our research question. Similar exploratory WGCNA approaches have been used in prior longitudinal studies (Baumgart et al., 2016, Cell Systems, Prebensen et al., 2023, Scientific Reports, Medina et al., 2024, eLife).

To properly account for repeated measures, we therefore applied mixed-effects models in all downstream analyses of module eigengenes, ensuring that inference on module-phenotype associations appropriately accounts for within-subject dependence. **We now clarify these points in the Methods (lines 894-902) and explicitly acknowledge the methodological limitations in the Discussion (lines 591-598). The module-phenotype association plot is replaced with the one calculated using mixed effects models in Figure 5b and Extended Data Figures 8a-b and we thank Peter Langfelder for helpful discussions in the Acknowledgements.**

- I am a bit concerned around the framing of the results of non-protein coding genes. Given the mRNA sampling protocol, this isn't exactly an unbiased sample of the non-coding transcriptome. I realize this is mentioned briefly in the discussion, however I am not sure it is fair to frame the results as characterizing properties of both coding and non-coding when only ~1/5th of them are represented.

We agree with the reviewer. We now refer to the transcript/gene subset captured by our protocol as "non-protein-coding (polyA+) genes/transcripts" or "polyadenylated non-protein-coding genes/transcripts". **We have updated the nomenclature consistently throughout the manuscript, including the Results and Figures**. All conclusions are now explicitly restricted to this subset. **We clearly define these genes based on the GENCODE annotation categories in the Methods (lines 671-674) and we state in the Limitations section of the Discussion (lines 584-590) that the method may capture approximately 20%-40% of all non-coding transcripts (Cheng et al, Science 2005; Yang et al., Genome Biology, 2011).**

- The methods write up around the analysis of infections is difficult to follow and it is unclear what was done. You have individuals with infections at some time points, and then you have matched controls, which makes sense. But then the samples from these same individuals during non-infectious timepoints are also used and it isn't clear to me how this is done. How are repeated measures handled here?

We agree that the original description of the infection analysis requires clarification. Briefly, matched control samples were only selected for samples collected during the acute phase of infection. Samples from non-infectious timepoints of individuals who experienced an infection at other visits were not used as controls. Gene modules of interest were then identified based on a combination of functional enrichment analysis and inspection of eigengenes in acute phase samples compared with their matched controls. Longitudinal samples from infected individuals outside the acute phase were only used for post hoc visualization of the module and gene expression trajectories over the course of infection and recovery and were not included in the statistical comparisons. Differences in cell proportions and hsCRP between control samples and longitudinal samples from infected individuals were tested using a Kruskal-Wallice test. We recognize that in this case a different approach is warranted to account for repeated measures and therefore **we have limited the test to only compare acute phase samples and matched controls** to avoid violating independence assumptions. Additionally, **we have adjusted the wording in the Results (lines 402-403), the Methods (lines 920-925), and corresponding Figures (Extended Data Fig. 8g) to clarify the general use of longitudinal samples in the infection analysis.**

Other comments/questions:
- The claim on paige 8 that heritable genes with higher inter-individual variability suggest environmental factors seems a bit strong given that there are several environmental factors measured here and most variance still seems to be in the residuals, suggesting random noise or inadequate power. Could an analysis be done to further support this claim?

We thank the reviewer for this comment and would like to clarify a misunderstanding. The manuscript refers to heritable genes exhibiting higher intra-individual variability, not higher inter-individual variability. We agree with the reviewer that attributing higher intra-individual variability in heritable genes specifically to environmental factors is not sufficiently supported by the current analyses. Since the residual variance may reflect a combination of technical noise, stochastic effects, or unmeasured biological and environmental influences, **we have hence tempered the language in the manuscript to avoid overinterpretation (lines 295-297).**

- The analysis corresponding to Fig 6 is, to me, perhaps the most interesting result here. Is this related to so-called "frequent DEGs" that come up across many studies? Are these more or less heritable than the others? Could this variation be impacting eQTL studies as well?

We are thankful for this excellent suggestion. We have now explicitly compared our "outlier genes" (genes highlighted in Fig. 6) with the published catalogue of "frequent DEGs" from Crow et al. (PNAS, 2019) and assessed enrichment. Frequent DEGs were significantly enriched in our outlier genes (Fig. 6g, Extended Data Fig. 9g), indicating substantial overlap between these gene sets, suggesting that recurrent different expression signals across studies may, in part, reflect underlying temporal variability rather than true trait associations.

We further investigated the heritability and eQTL characteristics of the “outlier genes”. “Outlier genes” showed significantly reduced eQTL discovery rates and heritability relative to all other genes (Extended Data Fig. 9e-f), suggesting that their apparent elevated inter-individual variability is not driven by genetic regulation.

These results have been included in the Result section “Impact of intra-individual variability on the accuracy of cohort-based transcriptomic studies” (lines 447-453 and lines 460-471), Methods (lines 951-971), and Figures 6g and Extended Data Fig. 9e-g.

- If possible, I would strongly prefer to see anonymized transcript quantification results uploaded to a data sharing platform such as Zenoodo for FigShare, rather than raw reads uploaded to a read archive available on “reasonable request”. This would enable reproduction of the results and reduce the barrier for further analyses.

We thank reviewer for this comment. Due to data-protection laws we are not allowed to share the data publicly. However, we agree that providing processed expression counts will simplify usage of the data. Therefore, we have now additionally included transcript- and gene-level expression counts used in our analysis in the EGA repository under restricted access.

Minor comments:
- The text in every figure is too small

We thank the reviewer for this comment and have increased the font size within the limits of the journal’s formatting guidelines (5-8pt). Specifically, we have increased the size of axis and legend labels from 5pt to 6pt, and the size of axis titles from 7pt to 8pt.

- I’m unclear on the difference between Figure 1c and ED2b

Fig. 1c (now Fig. 1e) shows the full distribution of the variance explained across individual genes capturing gene-level heterogeneity, whereas Extended Data Fig. 2b shows the average contribution of each parameter across genes. We have clarified this distinction in the figure legends.

Reviewer #2 (Remarks on code availability):

Scripts corresponding to major analyses seem to be present in the repository. As the data are only available on reasonable request, replication of the results or testing the code is not straightforward.

See comment above.
